# Genome-Wide Analysis of Major Facilitator Superfamily and Its Expression in Response of Poplar to *Fusarium oxysporum*

**DOI:** 10.3389/fgene.2021.769888

**Published:** 2021-10-22

**Authors:** Jian Diao, Shuxuan Li, Ling Ma, Ping Zhang, Jianyang Bai, Jiaqi Wang, Xiaoqian Ma, Wei Ma

**Affiliations:** ^1^ College of Forestry, Northeast Forestry University, Harbin, China; ^2^ Institute of Forest Protection, Heilongjiang Academy of Forestry, Harbin, China; ^3^ College of Medicine, Heilongjiang University of Chinese Medicine, Harbin, China

**Keywords:** *Populus davidiana* × *P. alba* var. *pyramidalis* Louche, *Fusarium oxysporum*, major facilitator superfamily, expression patterns, tissue-differential expression

## Abstract

The major facilitator superfamily (MFS) is one of the largest known membrane transporter families. MFSs are involved in many essential functions, but studies on the MFS family in poplar have not yet been reported. Here, we identified 41 *MFS* genes from *Populus trichocarpa* (*PtrMFSs*). We built a phylogenetic tree, which clearly divided members of PtrMFS into six groups with specific gene structures and protein motifs/domains. The promoter regions contain various cis-acting elements involved in stress and hormone responsiveness. Genes derived from segmental duplication events are unevenly distributed in 17 poplar chromosomes. Collinearity analysis showed that *PtrMFS* genes are conserved and homologous to corresponding genes from four other species. Transcriptome data indicated that 40 poplar *MFS* genes were differentially expressed when treated with *Fusarium oxysporum*. Co-expression networks and gene function annotations of *MFS* genes showed that *MFS* genes tightly co-regulated and closely related in function of transmembrane transport. Taken together, we systematically analyzed structure and function of genes and proteins in the PtrMFS family. Evidence indicated that poplar *MFS* genes play key roles in plant development and response to a biological stressor.

## Introduction

The major facilitator superfamily (MFS) is one of the largest membrane transporter families currently known (Chen et al., 2019). MFS’s diverse members are found in essentially all organisms in the biosphere. According to membrane transporter database TransportDB 2.0 (http://www.membranetransport.org/transportDB2/index.html) (Elbourne et al., 2017), the *Arabidopsis thaliana* MFS accounts for 128 transporters. María Niño-González proposed that MFS consists of 218 members, clustered in 22 families (María et al., 2019). Setyowati T. Utamia identifified 177 putative MFS transporters and classifified them into 17 subfamilies in *Penicillium marneffei* (Setyowati et al., 2020). Martin Broberg identifed 232 MFS transporters in maize pathogen *Cochliobolus heterostrophus* (Martin et al., 2021). MFS transporters belong to a wide family and in *Penicillium digitatum* more than 80 MFS have been identified due to the availability of the *P. digitatum* genome (Marcet-Houben et al., 2012). The MFS was first characterized in 1993, when a large class of transporters with 12 transmembrane helix domains were discovered among many membrane proteins (Marger and Saier, 1993). The function of MFS is involved in many essential functions. The basic function of members of this family is to assist in transporting substances across membranes (Bagchi et al., 2020). MFS proteins can transport many small molecules, such as monosaccharides, polysaccharides, amino acids, peptides, vitamins, enzyme cofactors, drug molecules, chromophores, and nucleotide bases (Lorca et al., 2007; Chen et al., 2008; Ming et al., 2010; Saier and Paulsen, 2011). Some MFS proteins are also closely related to immunological processes, such as virus invasion and pathogenic resistance (Nicolas et al., 2005).

Based on protein functions and sequence homology, the MFS can be divided into 89 families (Saier et al., 2009). Presently, more than 10,000 membrane protein genes from different species have been defined in the MFS. Most members of the MFS are ∼400–600 amino acids in length (Chun et al., 2018). Both the N-termini and C-termini of proteins are located within cells. Secondary structures of MFS proteins mainly comprise 12 *α*-helix transmembrane domains, which are divided into N-terminal domains and C-terminal domains, each of which contains six *α*-helices (Lee, 2015). In addition to transmembrane domains, some proteins in the MFS also have intracellular domains (Lu et al., 2010), which are conserved in the superfamily and perform important functions.

MFS transporters can confer resistance to a variety of toxic compounds, including specialized metabolites, fungicidal substances, and antibiotics (Sorbo et al., 2000; Fluman et al., 2012; Zhang et al., 2020). They can be used as drug H+ antiporters in microorganisms to indirectly regulate internal pH and stress response mechanisms in fungi (Santos et al., 2014); thus they can confer a multi-drug resistance (MDR) phenotype to fungi (Omrane et al., 2015). For example, it was reported that *MFS1* in Penicillium digitatum (*PdMFS1*) was able to confer the MDR phenotype due to efflux of fungicide (Ramon et al., 2019). Another study found that *PdMFS2* and *PdMFS3* could contribute to fungicidal resistance (Sorbo et al., 2000). The MFS transporter *Acinetobacter baumannii Fosfomycin efflux* (AbaF) actively effluxes fosfomycin, making *A. baumannii* resistant to this antibiotic (Atin et al., 2017). The expression level of AbaF is upregulated under fosfomycin exposure. AbaF participates in the secretion of biofilm matrix, which promotes the generation of bacterial pathogenicity and participates in expulsion of host defense molecules, leading to a significant impact on the virulence of *A. baumannii* (Atin et al., 2017). Most MFS transporters implicated in MDR belong to the drug:H(+) antiporter family 1 (DHA1) subfamily (Paulsen et al., 1996). DHA1-subfamily transporters of *Penicillium marneffei* provide resistance to various drugs, including azoles, polyene and antimalarial (Utami et al., 2020). TetA is an MFS pump which causes tetracycline resistance, and it is one of the most well-known antibiotic resistance mechanisms (Reisz et al., 2013; Grossman, 2016). In phytopathogenic fungi, MFS transporters can pump + to increase fungal invasiveness to host plants (Hayashi et al., 2002; Mathias et al., 2007; Ramin et al., 2007; Matthias et al., 2009). MDR has been found in a variety of phytopathogenic fungi, such as *Zymoseptoria tritici* (the pathogen of septoria leaf blotch on wheat) (Omrane et al., 2017), *P. digitatum* (the pathogen of green mold on citrus) (Wang et al., 2012; Wu et al., 2016; Ramon et al., 2019), and *Botrytis cinerea* (Matthias et al., 2009). The expression of *MFS1* in *Zymoseptoria tritici* is related to antifungal resistance (Omrane et al., 2015; Omrane et al., 2017). The expression of *MFS19* and *MFS54* play an important role in the oxidative stress response, the tolerance of xenobiotics such as fungicides, and the virulence of *Alternaria alternata* (Chen et al., 2017; Lin et al., 2018). Overexpression of *MFS1* in *P. digitatum* could make citrus more resistant to fungicides (Ramon et al., 2019). The MFS transporter mfsG is an important factor in determining the virulence effect of *B. cinerea* on *Brassicaceae* such as *Arabidopsis thaliana* (Vela-Corcía et al., 2019). MFS transporters help cells to better handle carbon sources and transport nutrients (especially sugars) to cells, which can provide advantages for the development of fungi (Ramó n-Carbonell and S á nchez-Torres, 2017). During the development of pathogens such as *Colletotrichum* and *Botrytis*, MFS transporters are responsible for the uptake of sugar in the form of glucose, mannose and fructose from environments (Monalessa et al., 2013; Vela-Corcía et al., 2019).

After host invasion, plant pathogens encounter potent plant defense compounds. MFS transporters may transport defense compounds and toxins secreted by pathogens out of host cells, thereby promoting plant resistance to pathogens. In this study, we performed systematic investigation of 41 *PtrMFS* genes, studying structures and functions of *PtrMFS* genes and proteins, respectively, as well as phylogenetic relationships, cis-acting elements, chromosomal distribution, collinearity across related species, and duplication events. Additionally, we analyzed transcriptome data to identify differentially expressed poplar *MFS* genes during *Fusarium oxysporum* infection. Finally, we performed a co-expression network analysis of poplar *MFS* genes, and gene set enrichment analysis revealed pathways related to a variety of biological processes. This research provides novel characterization of poplar *MFS* genes and establishes a theoretical basis for functional verification.

## Materials and Methods

### Identification of MFS Proteins in *Populus trichocarpa*

Genome data for *P. trichocarpa* were downloaded from Phytozome v12.1 (https://phytozome.jgi.doe.gov/pz/portal.html) (Goodstein et al., 2012). The typical MFS protein domains (PF07690, PF16983, PF05631, PF07672, PF05977, PF06779, PF12832, and PF13347) were obtained from Pfam (http://pfam.xfam.org/) (Finn et al., 2014). Scanning the poplar genome for potential PtrMFS proteins was conducted with HMMER3 (Jaina et al., 2013). Verification was performed using the SMART database (http://smart.embl-heidelberg.de/) (Ivica and Peer, 2018) and Pfam database to remove proteins without MFS domains.

### Phylogenetic Relationship and Physicochemical Properties of MFS Proteins

MFS protein sequences of *P. trichocarpa* and *A. thaliana* were downloaded from Phytozome. We used MEGA v5.1 (Tamura et al., 2011) with the Maximum-Likelihood (ML) method to construct a phylogenetic tree of MFS proteins using the JTT (protein mutation data matrix) + G (site-specific variations in mutation rate) + F (mutation frequency data) model.

Physical and chemical properties of PtrMFS proteins were predicted with ProtParam (https://web.expasy.org/protparam/) (Gasteiger et al., 2003), including the number of amino acids, molecular weight, theoretical isoelectric point (pI), aliphatic index, grand average of hydrophilicity (GRAVY), chemical formulas, total number of atoms, and instability index.

### Gene Structure and Protein Motif Analysis of PtrMFS Family

To analyze gene structures of *PtrMFSs*, we downloaded DNA and coding sequences of PtrMFS from Phytozome database. DNA and coding sequences for each *PtrMFS* gene were aligned to obtain gene structures. TBtools (Chen et al., 2018) was used to visualize gene structures of *PtrMFSs*, and MEME (http://meme-suite.org/) (Bailey et al., 2006) was used to identify conserved motifs in PtrMFS proteins. Annotations of identified motifs were obtained from InterProScan (https://www.ebi.ac.uk/interpro/search/sequence/) (Quevillon et al., 2005).

### Secondary and Tertiary Structures Prediction of PtrMFS Proteins

Secondary structures of PtrMFS proteins were predicted by SOPMA (https://npsa-prabi.ibcp.fr/cgi-bin/npsa_automat.pl?page=npsa_sopma.html) (Geourjon and Delé age, 1995), and tertiary structures were constructed by SWISS-MODEL (https://swissmodel.expasy.org/) (Torsten et al., 2003).

### Topological Heterogeneity Model and Subcellular Localization Prediction

Topological heterogeneity models of PtrMFS proteins were predicted with Protter (http://wlab.ethz.ch/protter/start/) (Ulrich et al., 2014). Subcellular localization was predicted using WoLF PSORT (https://wolfpsort.hgc.jp) (Paul et al., 2007).

### Cis-Acting Elements Analysis

For each *PtrMFS* gene, sequences starting at 2,000 bp upstream of the start codon were downloaded from Phytozome. Cis-acting elements were extracted using PlantCARE (http://bioinformatics.psb.ugent.be/webtools/plantcare/html/) (Rombauts et al., 1999). TBtools was used to visualize cis-acting elements.

### Chromosome Distribution and Collinearity Analysis

*PtrMFS* genes were mapped to the genome of *P. trichocarpa*, and the chromosomal distribution of *PtrMFSs* in poplar was visualized with TBtools. TBtools and MCScanX (Wang et al., 2012) were used to analyze tandem duplication events in the *PtrMFS* gene family, and Dual Synteny Plotter (Tang et al., 2008) was used to analyze segmental duplication events and collinearity between *PtrMFSs* and homologous gene pairs from other species (*A. thaliana*, *Eucalyptus grandis*, *Oryza sativa*, and *Solanum lycopersicum*). TBtools was used to visualize the results. The ratio of non-synonymous substitution to synonymous substitution (*Ka/Ks*) of duplicate gene pairs was determined with KaKs_Calculator (Zhang et al., 2006).

### Sample Preparation

WT Pdpap seedlings used in this research were cultured on 0.5X Murashige and Skoog (MS) medium supplemented with 0.01 mg/ml 1-naphthaleneacetic acid (NAA). Two-month-old WT Pdpap plants at the same growth stage was selected. Fifty milliliters of *F. oxysporum* at a spore density of 1 × 10^5^/ml was poured onto Pdpap roots (Kang et al., 2018). Infection times were 6, 12, 24, or 48 h. WT Pdpap treated with 50 ml ddH_2_O was used as control. Every treatment consisted of 3 duplicated samples. The materials involved in this research and their preparation methods have been reported in detail in our previous research (Diao et al., 2021).

### Gene Expression Analysis

RNA-Seq was used to explore gene expression patterns of the *MFS* gene family from PdPap under *F. oxysporum* stress. The data were described in detail in our previous research (Diao et al., 2021). Differentially expressed genes (DEGs) were identified with DESeq2 using log_2_ (fold change) ≥ and adjusted *p*-value (padj) ≤ 0.05 as the criteria. Expression patterns of *PdPapMFS* genes under different treatments of *F. oxysporum* at 6, 12, 24, and 48 h were visualized with heatmaps. We identified upregulated and downregulated DEGs in response to *F. oxysporum* and displayed the data by Venn diagrams. To validate the RNA-Seq data, we further analyzed expression levels of DEGs under *F. oxysporum* stress by qRT-PCR. The qRT-PCR was performed on a Stratagene Mx3000P real-time PCR system (Agilent Technologies, Santa Clara, CA, United States) using the 2 × SYBR Green qPCR Master Mix kit (Bimake, Shanghai, China). Reaction systems are shown in Supplementary Table S1 and primer sequences designed by Primer5.0 (Zhai et al., 2008) are shown in Supplementary Table S2. qRT-PCR amplification conditions were as follows: initial denaturation at 94°C for 30 s; 44 cycles of 94°C for 12 s, 58°C for 30 s, and 72°C for 45 s, then 79°C for 1 s. The reaction specificity was determined by performing a melting-curve analysis from 55°C to 99°C, with fluorescence readings taken every 0.5°C for 1 s. The amplification curve was obtained after analyzing the raw data, and the cycle threshold (Ct) was set with a fluorescence threshold of 0.01 (Wang et al., 2012). Relative expression level of target genes was calculated by the 2^−△△Ct^ method (Kenneth and Thomas, 2002). Three duplicates were set for each gene. *PdpapActin* and *PdPapEF1-α* were used as the internal control genes (Huang et al., 2008).

### Gene Co-Expression Analysis and Gene Ontology Annotation

We analyzed co-expression-based gene networks using STRING (Damian et al., 2011), and visualized the results with Cytoscape (Ideker, 2011). Co-expression analysis was performed on the 41 *PdPapMFS* genes identified from RNA-Seq data analysis as described above. Genes were annotated with gene ontology (GO)-based functions using agriGO v2.0 (http://systemsbiology.cau.edu.cn/agriGOv2/index.php) (Du et al., 2010).

### Statistical Analysis

Data were analyzed with the Statistical Software Package for Social Science (SPSS) version 17.0 (Kumar, 2014). Using Student’s t-test to compare the data, *p* < 0.05 was considered statistically significant (Choi et al., 2012). Significant differences (*p* < 0.05) are indicated by different lowercase letters in figures.

## Results

### Phylogenetic Relationship and Physicochemical Properties of PtrMFSs

**FIGURE 1:**
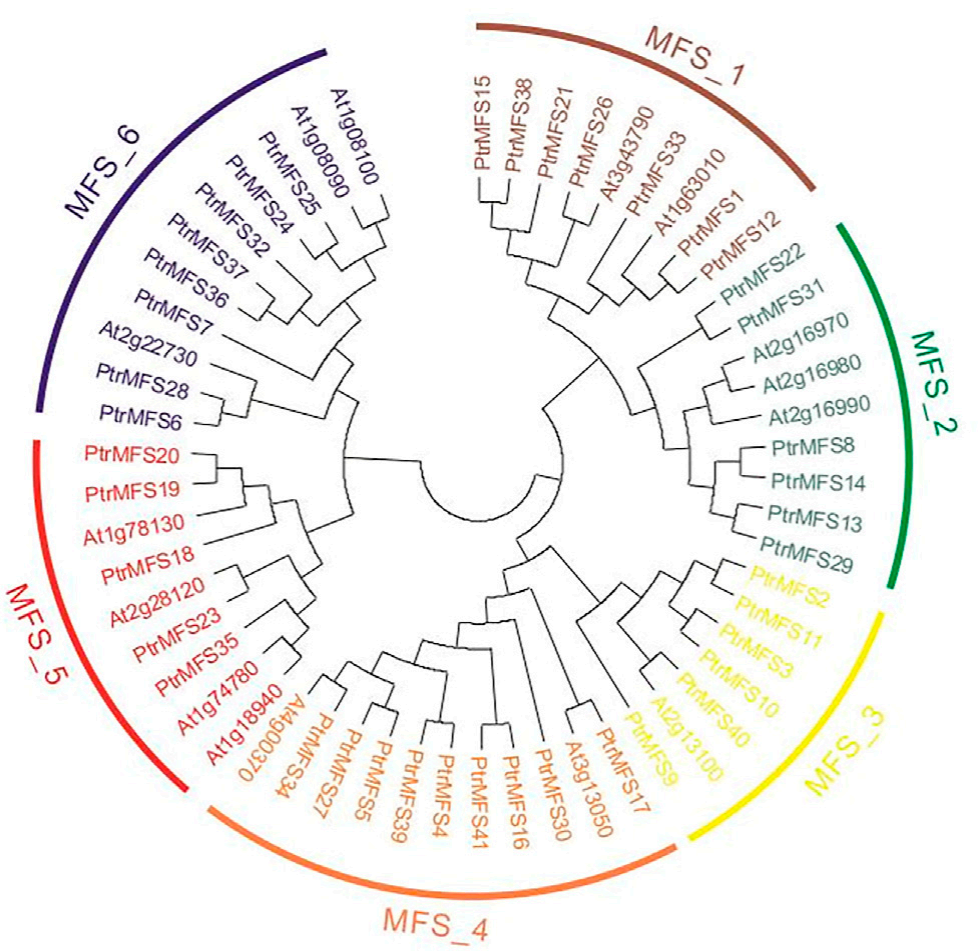
Phylogenetic analysis of MFS proteins in poplar and Arabidopsis. The dendrogram of 56 MFS proteins was performed by MEGA5 with the ML method based on JTT + G + F model. Different groups are marked with different colors.

We identified 41 *MFS* genes in *P. trichocarpa* (each named *PtrMFS* with a number based on position in the poplar genome; Supplementary Table S3). To determine evolutionary relationships of genes in this family, we constructed a ML phylogenetic tree (Figure 1) using protein sequences from poplar and Arabidopsis. As shown in Figure 1, we classified *PtrMFSs* into six groups of various sizes. Group MFS_4 is the largest with nine genes, while group MFS_5 is the smallest with five genes.

The physicochemical properties of the identified PtrMFSs vary significantly (Supplementary Table S3). The average length of PtrMFS proteins is 498 amino acids (range = 372–698 aa) and the average molecular weight is 54.27 kDa (range = 40.20–78.23 kDa). The theoretical pI of PtrMFSs is in the range of 5.54–10.07 and the aliphatic index is in the range of 85.96–118.46. The hydrophilicity value of PtrMFSs ranges from 0.183 to 0.758. All of the proteins are predicted to be hydrophilic. PtrMFSs consist of five elements: C, H, N, O, and S. The total number of atoms in each protein range from 5,724 to 11,110. The instability indexes of PtrMFSs range from 24.80 to 52.06; an index >40 indicates an unstable protein, while <40 indicates a stable protein.

### Sequence Structure Analysis of PtrMFSs

**FIGURE 2:**
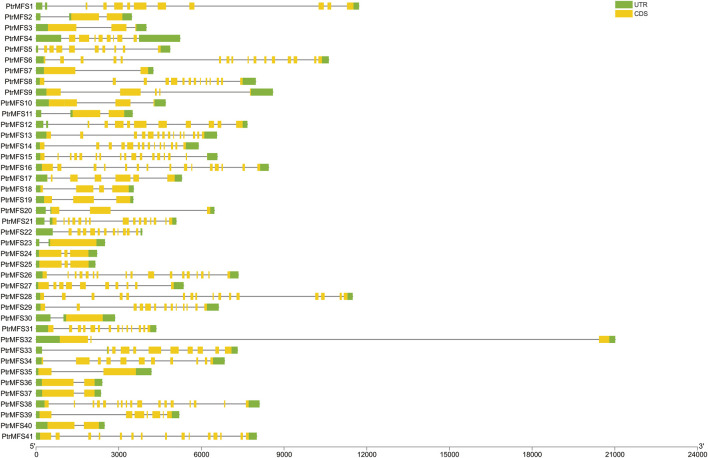
DNA structures of *PtrMFS* gene family in poplar. Green boxes represent untranslated regions. Yellow boxes denote coding regions. Black lines indicate introns.

*PtrMFS* genes within the same groups share similar structures with respect to introns and exons (Figure 2). Four members in group MFS_2 contain 14 exons. Three members in group MFS_3 contain 2 exons and only 1 intron. Three members in group MFS_6 contain 2 exons and another three members contain 3 exons. Members with closer relationships share more similar gene structures and exon lengths.

**FIGURE 3:**
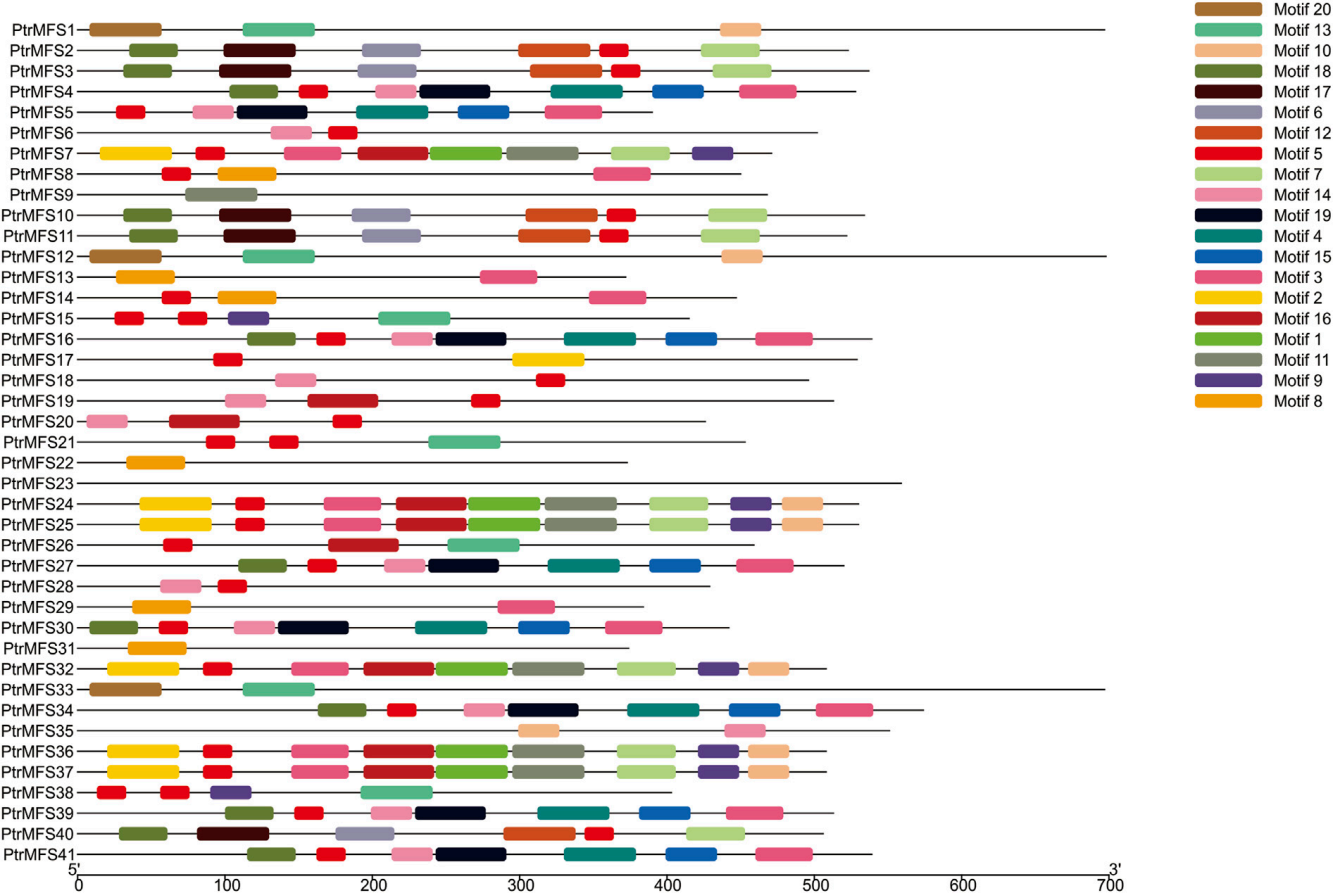
Protein motifs of PtrMFS family in poplar. Different colorful boxes represent different motifs.

PtrMFS proteins with similar evolutionary relationships have the same or similar conserved domains. We identified 20 conserved motifs using MEME, and motif annotations were predicted with Pfam and InterProScan (Supplementary Table S4). We found that motifs 2, 3, 4, 5, 6, 7, 8, 12, 13, 14, 17, and 19 were annotated as MFS transporters. Motifs 2, 3, 11, and 16 were high affinity nitrate transporter-related; motif 8 was tetracycline resistance domain. Motif 13 was annotated as protein zinc induced facilitator. Motif 15 was annotated as anion transporter and solute carrier family. Motif 1 was annotated as LytB protein and motif 20 was annotated as SPX domain. Motifs 9, 10, and 18 were unknown and could not be annotated. Results show that motifs 13 and 20 are present only in all members of group MFS_1 (Figure 3). Motif 8 is present only in all members of group MFS_2. Motif 6, 7, 12, 17, and 18 are present only in members of group MFS_3. Motif 4, 5, 14, 15, and 19 are present only in all members of group MFS_4. Motif 1, 2, 5, 7, 9, 11, and 16 only occurred in members of group MFS_6. These results demonstrate that there are many group-specific motifs, which may be correlated to specific biological functions.

### Secondary and Tertiary Structures of PtrMFSs

In the secondary structure of PtrMFS proteins (Supplementary Table S5), an average of 234.59 aa belonged to alpha helixes, accounting for 46.93% of total secondary structures. On average, a further 74.73 aa (15.08%) belonged to extended strands, 23.29 aa (4.71%) belonged to beta turns, and 165.37 aa (33.28%) belonged to random coils.

Tertiary structures were generated primarily using 6s4m.1.A and 6e9c.1.A as templates (Supplementary Table S6). They have functions of major facilitator superfamily domain-containing proteins. Sequence identity of the 41 PtrMFS proteins identified in this study and their corresponding templates is 20.80% (11.02–37.71%) on average.

### Topological Heterogeneity Models and Subcellular Localization of PtrMFSs

Topological heterogeneity models of PtrMFSs proteins (Supplementary Table S7) showed that all members of the PtrMFS family have transmembrane helical segments. PtrMFS19, PtrMFS23, and PtrMFS26 have no N-glycosylation sites. PtrMFS8, PtrMFS14, PtrMFS18, PtrMFS19, and PtrMFS28 have signal peptides.

Subcellular localization predictions (Supplementary Table S8) showed that a majority of PtrMFSs are located at the plasma membrane. PtrMFS26 is predicted to be located at the endoplasmic reticulum, PtrMFS31 at the vacuole, and PtrMFS4, PtrMFS5, PtrMFS13, PtrMFS22, PtrMFS29, PtrMFS39, and PtrMFS41 at the chloroplast.

### Cis-Acting Elements Analysis in Promoters of *PtrMFS* Genes

**FIGURE 4:**
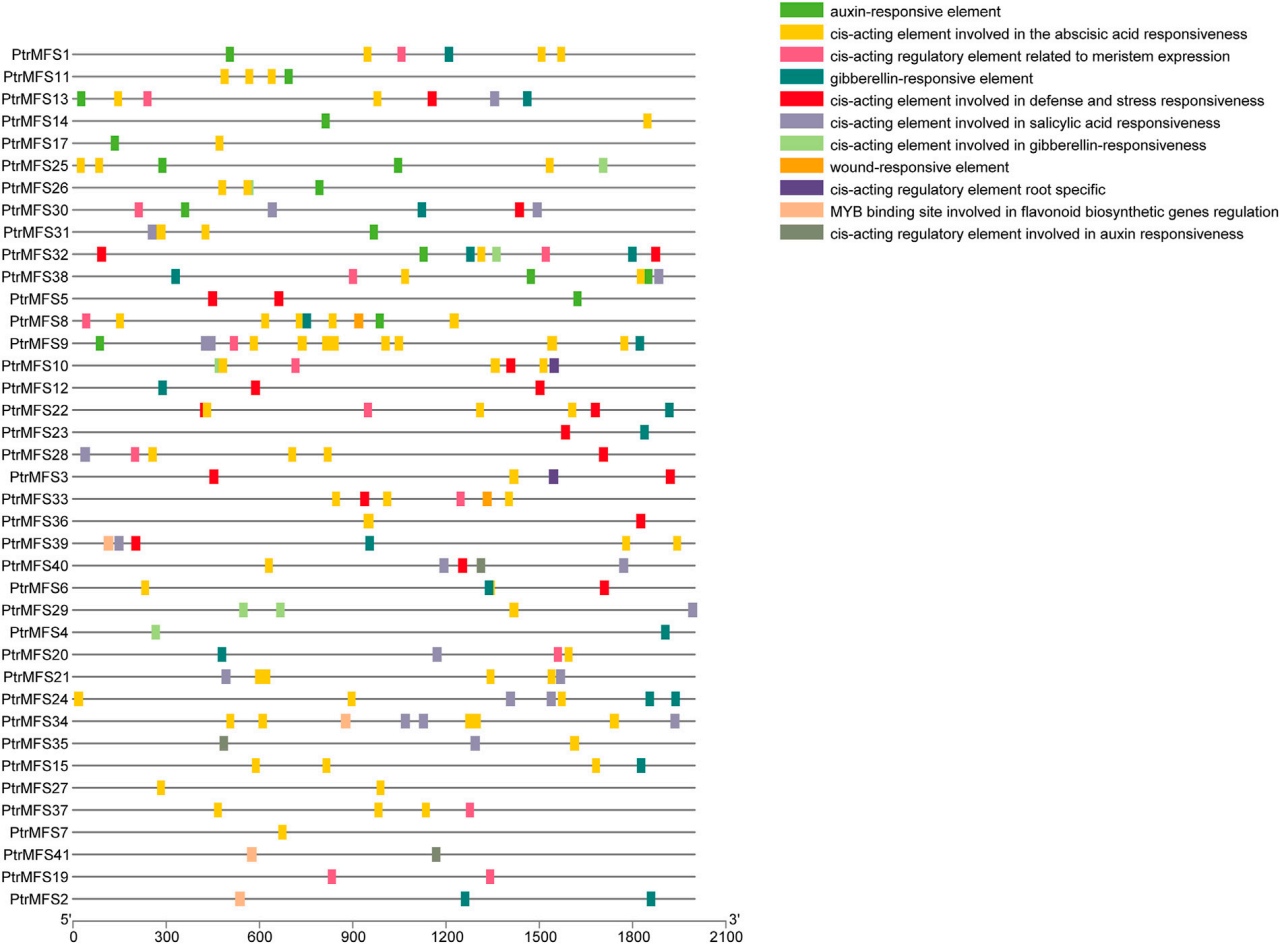
Analysis of cis-acting elements in promoters of *PtrMFS* genes. The patterns in different colors represent different cis-acting elements.

We used PlantCARE (http://bioinformatics.psb.ugent.be/webtools/plantcare/html/) to predict cis-acting elements within 2,000 bp upstream of *PtrMFS* genes (Supplementary Table S9; Figure 4). Many predicted cis-acting elements identified are involved in hormone responses, such as response to auxin, response to gibberellin, response to salicylic acid, and response to abscisic acid. Many elements are also predicted to be involved in defense and stress responses, meristem expression, root specific expression, flavonoid biosynthetic gene regulation, and wounding response.

### Chromosomal Location and Collinearity Analysis of PtrMFSs

**FIGURE 5:**
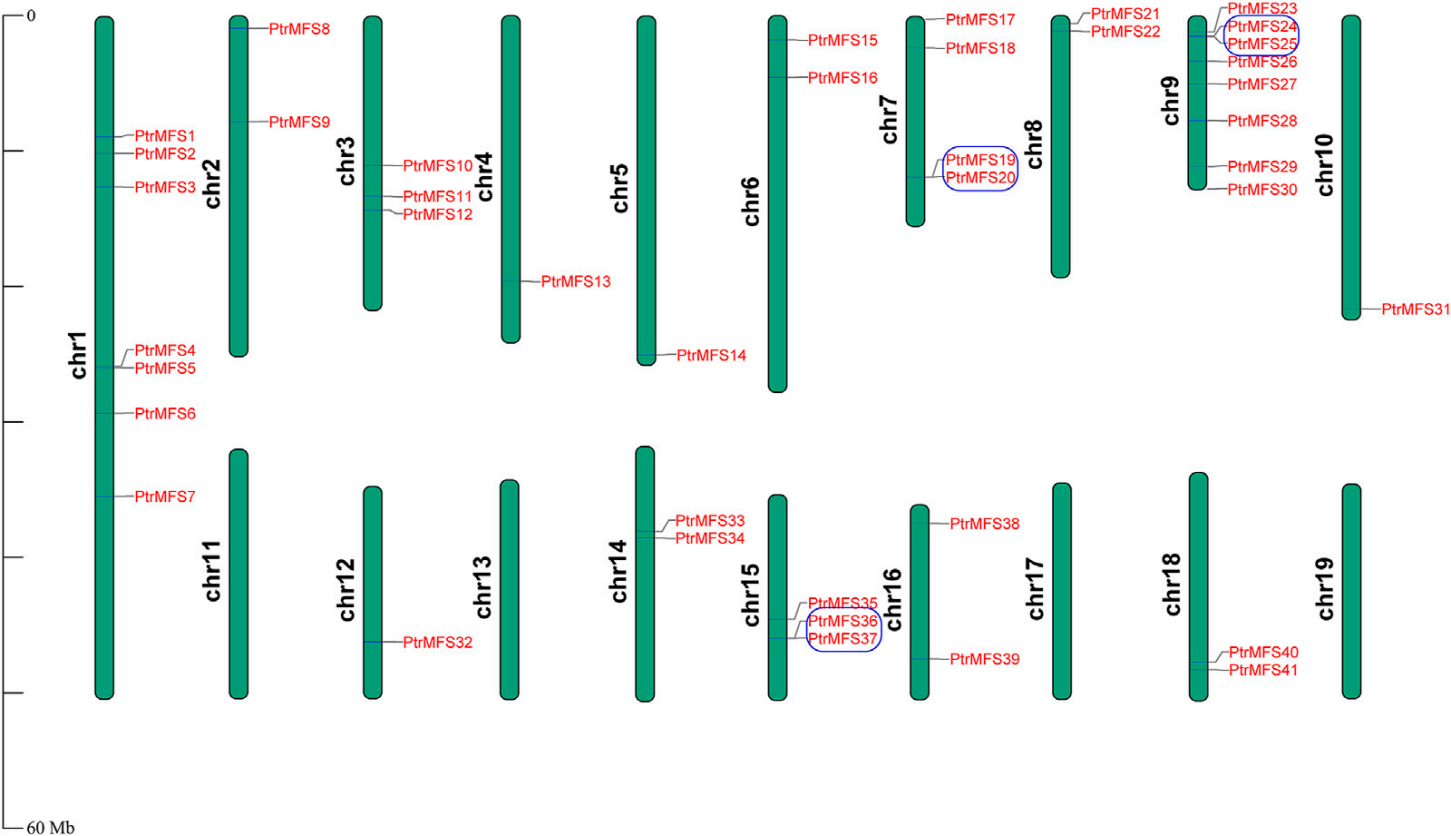
Chromosomal distribution of *PtrMFS* genes. Chr1–19 represent chromosome numbers 1–19. Blue boxes represent pairs of tandem repeated genes.

The location of *PtrMFS* genes within chromosomes is variable (Figure 5); they are unevenly distributed in 16 of the 19 poplar chromosomes. The number of genes located in each chromosome is irrelevant to the size of chromosomes. Chromosome 9 contains the most *PtrMFS* genes, with 8. Only one *PtrMFS* gene each was located on chromosome 4, chromosome 5, chromosome 10, and chromosome 12, and no *PtrMFS* genes were found on chromosome 11, chromosome 13, chromosome 17, or chromosome 19.

**FIGURE 6:**
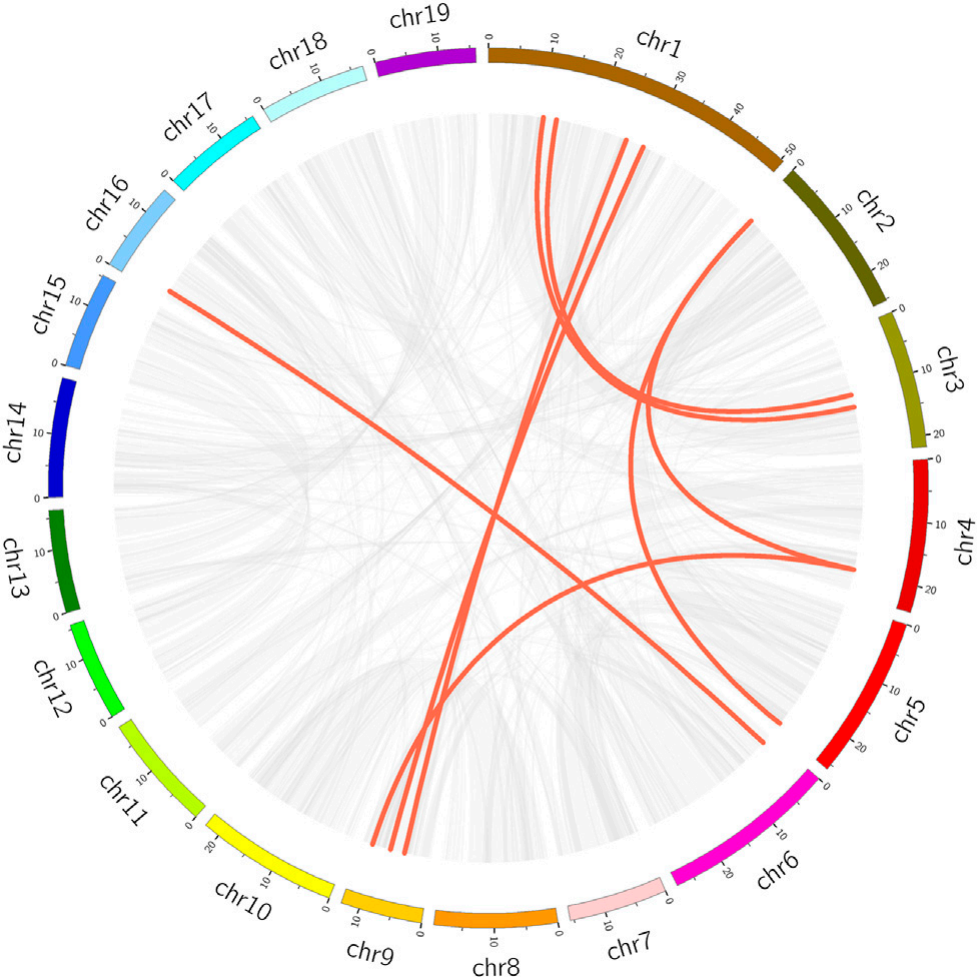
Segmental duplication events of *PtrMFS* genes. Chr1–19 are represented by different colorful rectangles. Red lines represent collinear pairs of *PtrMFS* genes. Gray lines indicate collinear pairs in all poplar genome.

We analyzed within-genome duplication events of *PtrMFS* genes using MCScanX. *PtrMFS19* and *PtrMFS20* (on chromosome 7), *PtrMFS24* and *PtrMFS25* (on chromosome 9), *PtrMFS36* and *PtrMFS37* (on chromosome 15) were categorized as derived from tandem duplication events. Fourteen genes exhibit 8 pairs of segmental duplication events (Figure 6; Supplementary Table S10). They are unevenly distributed in eight of 19 chromosomes. Segmental duplication events may be the important driving force for diversity of *PtrMFS* genes. The ratio of *Ka/Ks* is an important indicator of selective pressure in evolution, with a *Ka/Ks* < 1 indicating negative selection. *Ka/Ks* values of *PtrMFS* duplicate genes range from 0.16 to 0.37, with an average value of 0.24, suggesting that *PtrMFS* genes have been subject to purifying selection during evolution.

**FIGURE 7:**
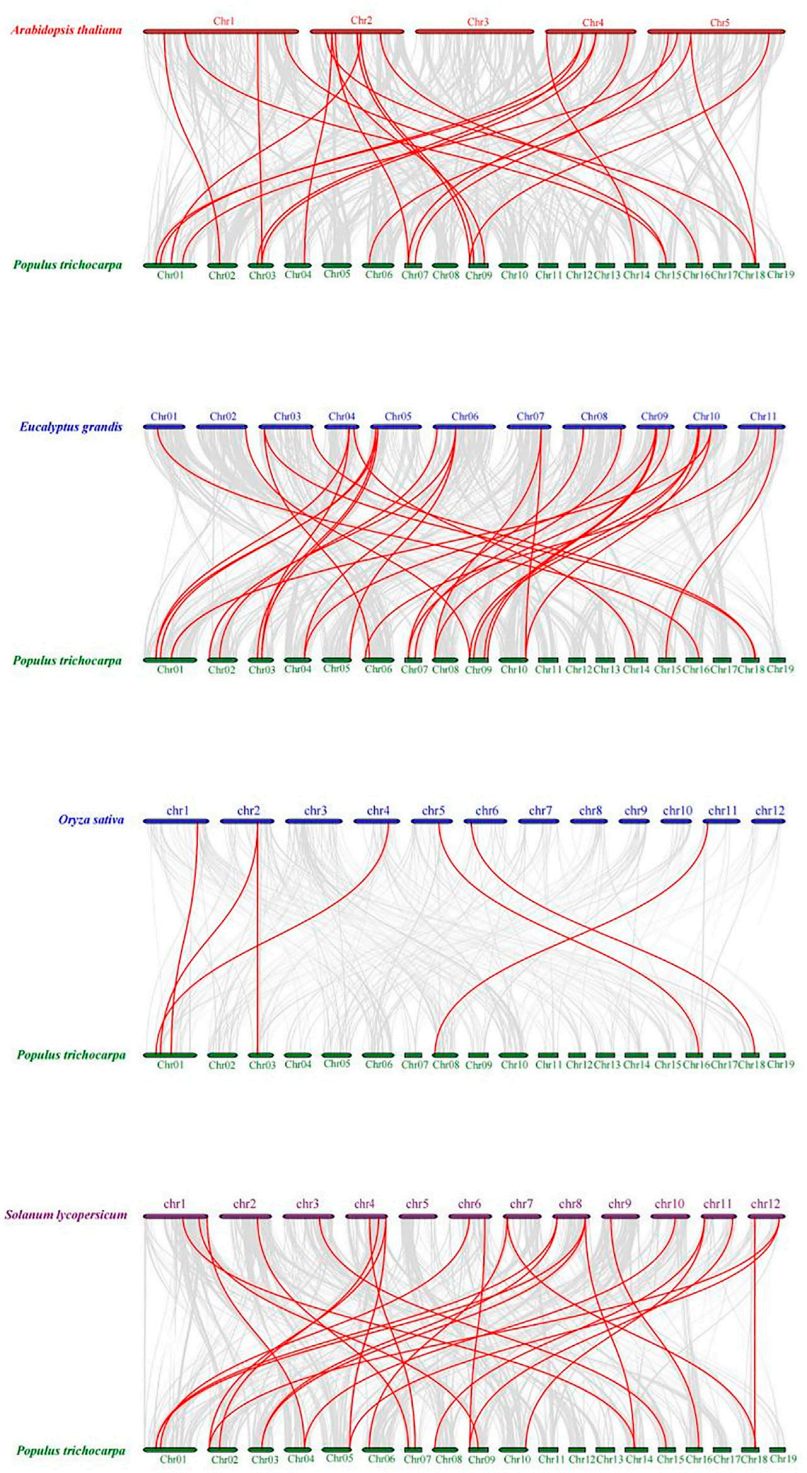
Collinearity relationship maps of *PtrMFS* genes in poplar to other four species. Red lines denote collinearity between *PtrMFS* genes and other species. Gray lines represent collinearity between poplar genome and that are orthologous to the other species.

To explore the DNA sequence similarity between *PtrMFS* genes and related genes from other representative species, we constructed collinearity relationship maps of *Populus trichocarpa* with three dicotyledons (*E. grandis*, *S. lycopersicum*, and *A. thaliana*) and one monocotyledon (*O. sativa*) (Figure 7). We identified 32 repetitive events in *E. grandis*, 26 in *S. lycopersicum*, 23 in *A. thaliana*, and seven in *O. sativa* (Supplementary Table S11). Collinearity blocks were mainly distributed in the first 10 chromosomes of *P. trichocarpa*. There was greater collinearity between *PtrMFS* genes and those in dicotyledons than those in the monocotyledon.

### *PtrMFS* Gene Expression in Response to *F. oxysporum*

**FIGURE 8:**
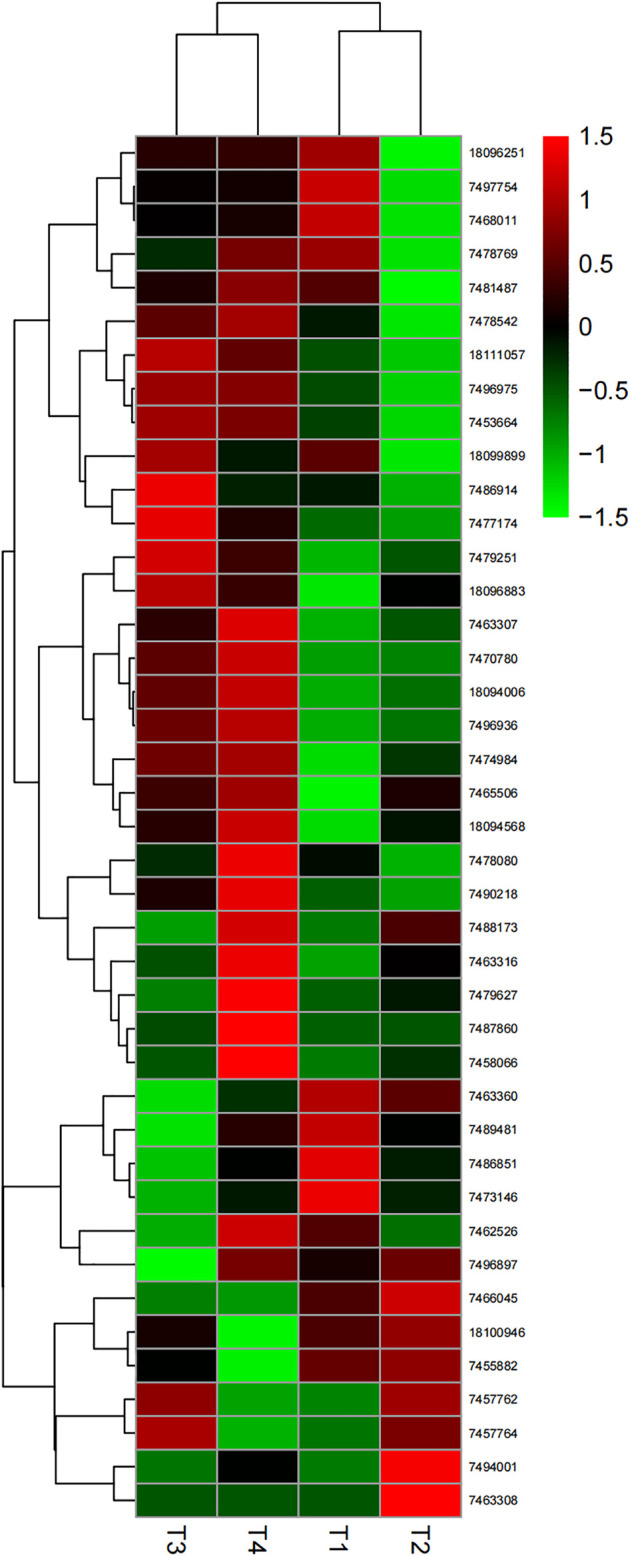
Heatmap of different expression levels in *PdpapMFS* genes under different treatments by *F. oxysporum*. Cluster analyses were based on log_2_FPKM. Red boxes represent highly expressed genes. Green boxes represent low expressed genes. The left side represents gene clusters. T1, T2, T3, and T4 indicated that PdPap were infected by *F. oxysporum* for 6, 12, 24, and 48 h.

We analyzed expression patterns of *PdPapMFS* genes in response to *F. oxysporum* with different lengths of time post-inoculation using RNA-Seq data (Supplementary Table S12). Statistical results indicated that *PdPapMFSs* expression are all in different fold-changes (Figure 8). The relative gene expression values of *PdPapMFS* are shown in Supplementary Table S13.

**FIGURE 9:**
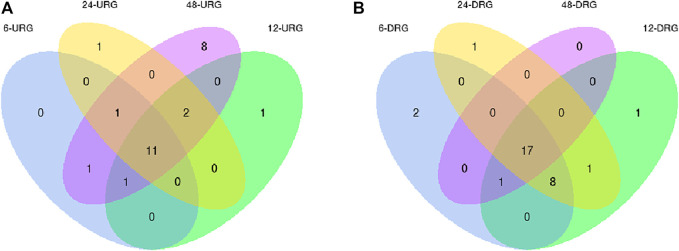
Venn diagrams of DEGs in response to *F. oxysporum* stress under different treatments. **(A)** Venn diagram of up-regulated DEGs (URGs) in response to *F. oxysporum* stress under different treatments. **(B)** Venn diagram of down-regulated DEGs (DRGs) in response to *F. oxysporum* stress under different treatments.

**FIGURE 10:**
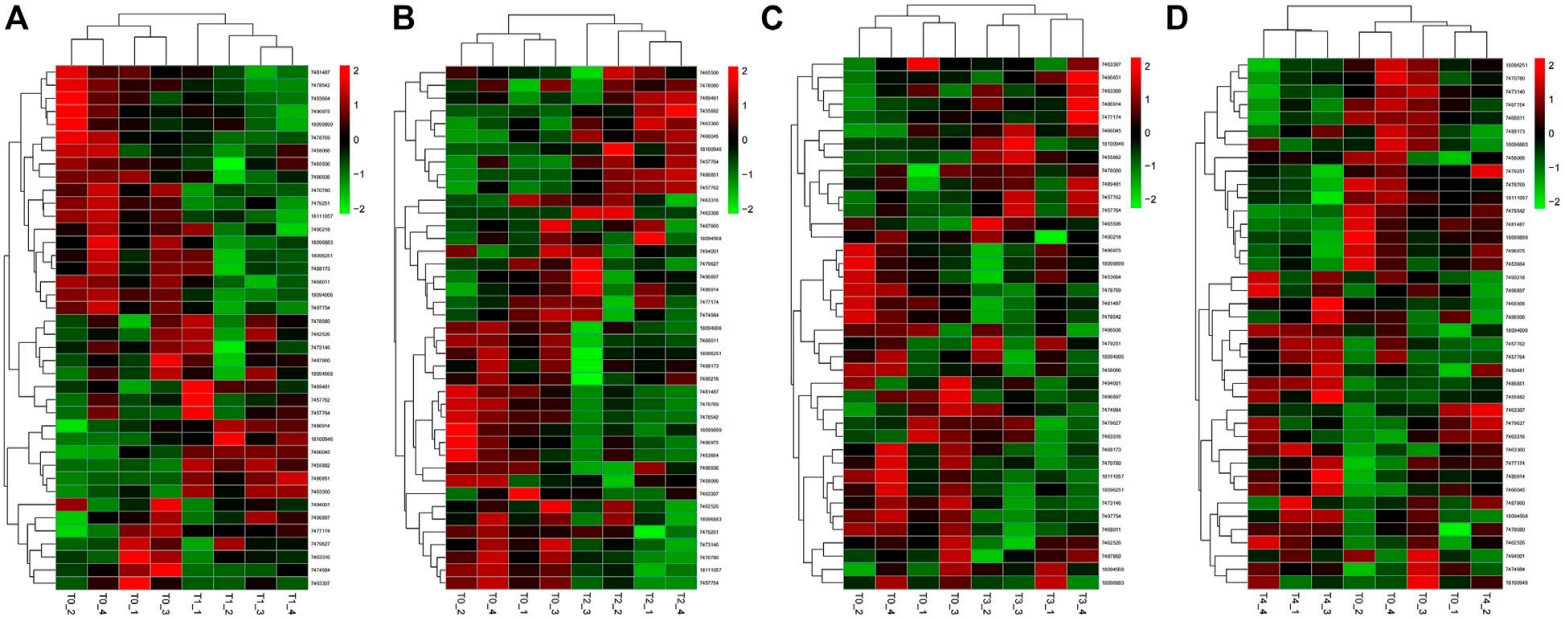
Heatmaps of DEGs in response to *F. oxysporum* stress under different treatments. **(A)** The heatmap of DEGs in response to *F. oxysporum* stress at 6 h. **(B)** The heatmap of DEGs in response to *F. oxysporum* stress at 12 h. **(C)** The heatmap of DEGs in response to *F. oxysporum* stress at 24 h. **(D)** The heatmap of DEGs in response to *F. oxysporum* stress at 48 h. Cluster analyses were based on log_2_FPKM. Red boxes represent highly expressed genes. Green boxes represent low expressed genes. The left side represents gene clusters. T1, T2, T3, and T4 indicated that PdPap were infected by *F. oxysporum* for 6, 12, 24, and 48 h.

In the samples collected 6 h after inoculation with *F. oxysporum*, we identified 40 DEGs, including 13 upregulated genes, and 27 downregulated genes. *PdPapMFS38* was the most upregulated (3.14X) and *PdPapMFS25* was the most downregulated (−2.07X). In the 12 h treatment group, 41 DEGs were found, including 14 upregulated genes and 27 downregulated genes. *PdPapMFS38* was the most upregulated (3.22X) and *PdPapMFS17* was the most downregulated (−1.43X). In the 24 h treatment group, 40 DEGs were found, including 14 upregulated genes, and 26 downregulated genes. *PdPapMFS38* was the most upregulated (2.93X) and *PdPapMFS17* was the most downregulated (−1.16X). In the 48 h treatment group, 40 DEGs were found, including 23 upregulated genes, and 17 downregulated genes. *PdPapMFS38* was the most upregulated (2.32X) and *PdPapMFS17* was the most downregulated (−1.14X). DEG expression in the four treatment groups was visualized with heatmaps (Figure 9) and Venn diagrams (Figure 10).

### Verification of *PtrMFS* Genes Expression by RT-qPCR

**FIGURE 11:**
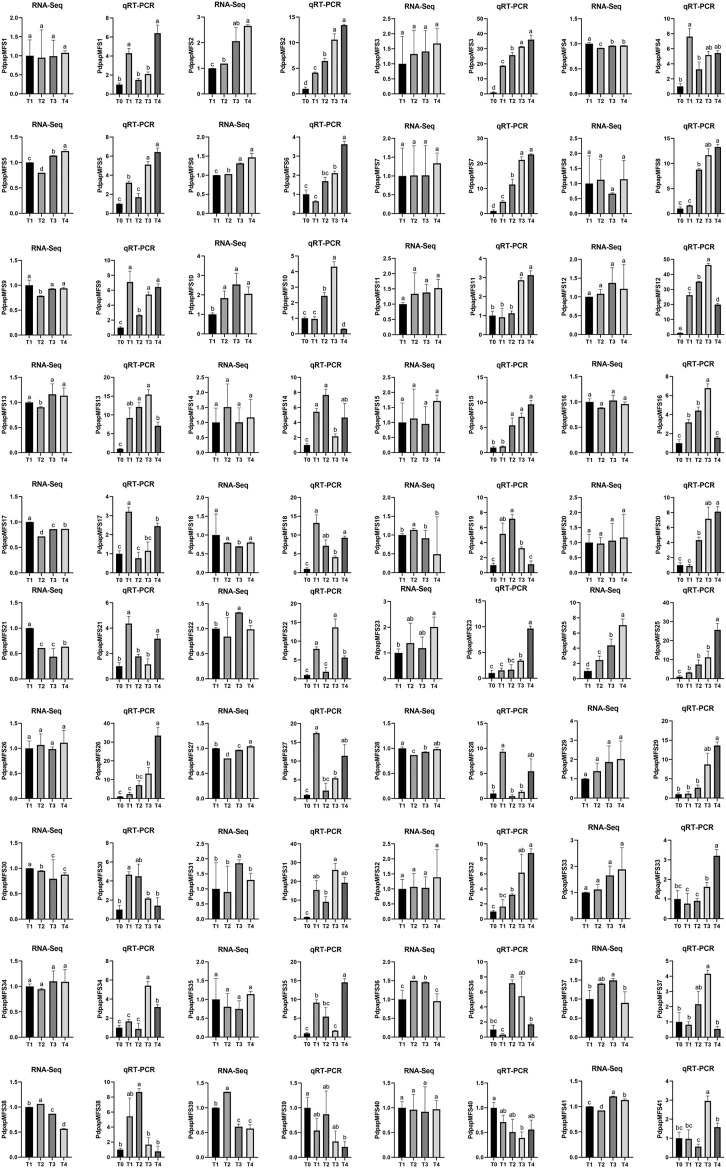
DEG expression levels in Pdpap based on RNA-Seq and qRT-PCR. Expression levels in RNA-Seq was quantified by fragments per kilo-bases per million mapped reads (FPKM). T0, T1, T2, T3, and T4 indicated that PdPap were infected by *F. oxysporum* for 0, 6, 12, 24, and 48 h. Error bars represented standard deviation of three independent replicates. Significant differences (*p* < 0.05) were indicated by different lowercase letters. Gene co-expression and gene ontology analysis.

To validate the RNA-Seq data, we performed qRT-PCR to analyze expression levels of putative DEGs in response to *F. oxysporum* stress. Results of RNA-Seq and qRT-PCR were generally consistent, with a few exceptions such as *PdPapMFS8*, *PdPapMFS16*, *PdPapMFS34*, which may be caused by experimental errors of RNA-Seq or qRT-PCR (Figure 11).

### Gene Co-Expression and Gene Ontology Analysis

**FIGURE 12:**
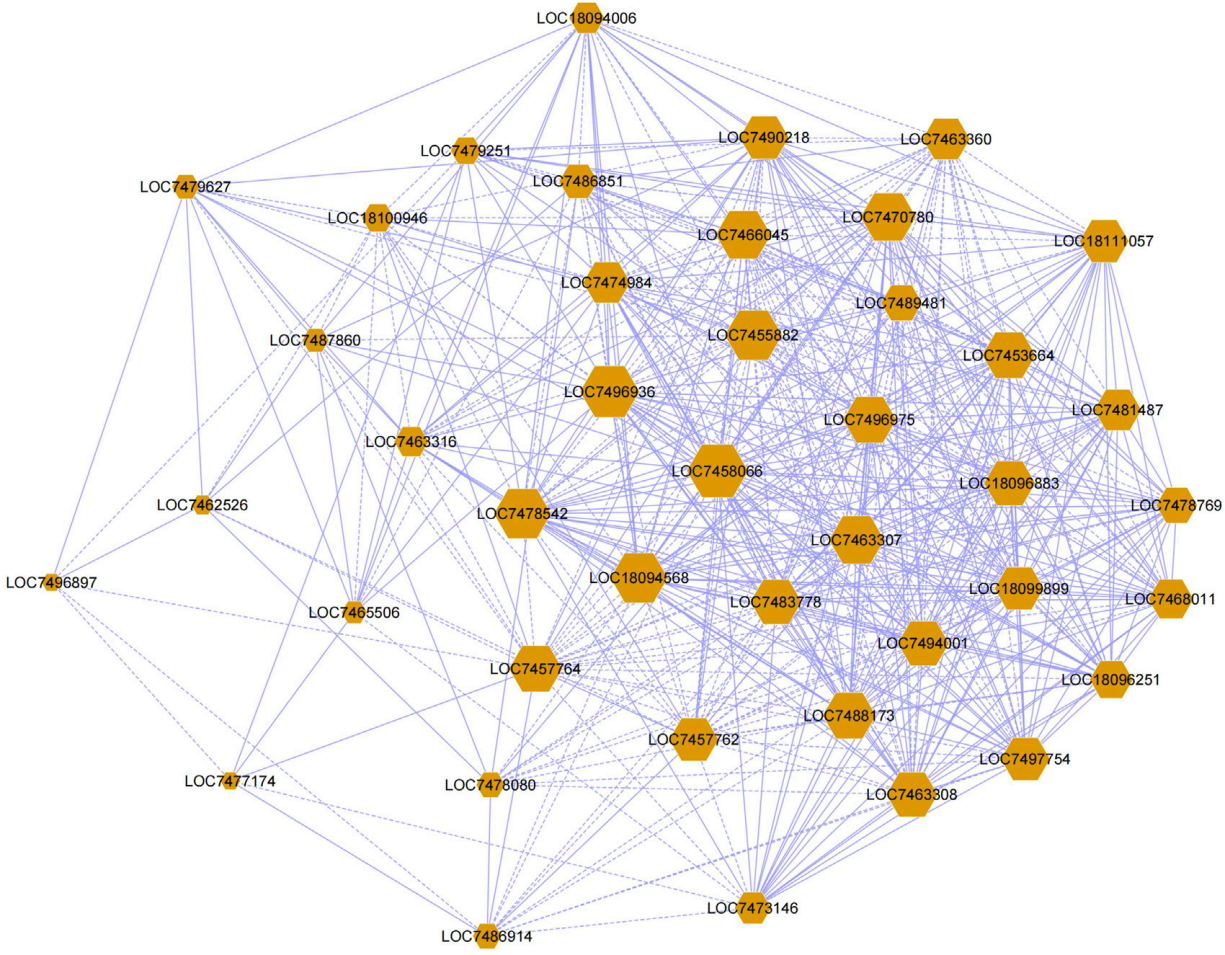
Co-expression-based gene networks of *PtrMFS* gene family. Dots represent genes. Lines indicate that they have co-expression relationships.

We constructed a co-expression network using RNA-Seq data of the 41 *PtrMFS* genes (Figure 12). A large proportion of the genes are shared in the networks, such as *PtrMFS32* (LOC7458066), *PtrMFS38* (LOC7455882), *PtrMFS33* (LOC7496936), *PtrMFS6* (LOC7470780), *PtrMFS5* (LOC7478542), *PtrMFS39* (LOC7466045), *PtrMFS3* (LOC18094568), *PtrMFS13* (LOC7453664), *PtrMFS25* (LOC7463307). We have observed that the shared gene expression patterns in the co-expression network are not exactly the same, which indicated that *MFS* genes under the stress of *F. oxysporum* may have complex regulatory characteristics.

**FIGURE 13:**
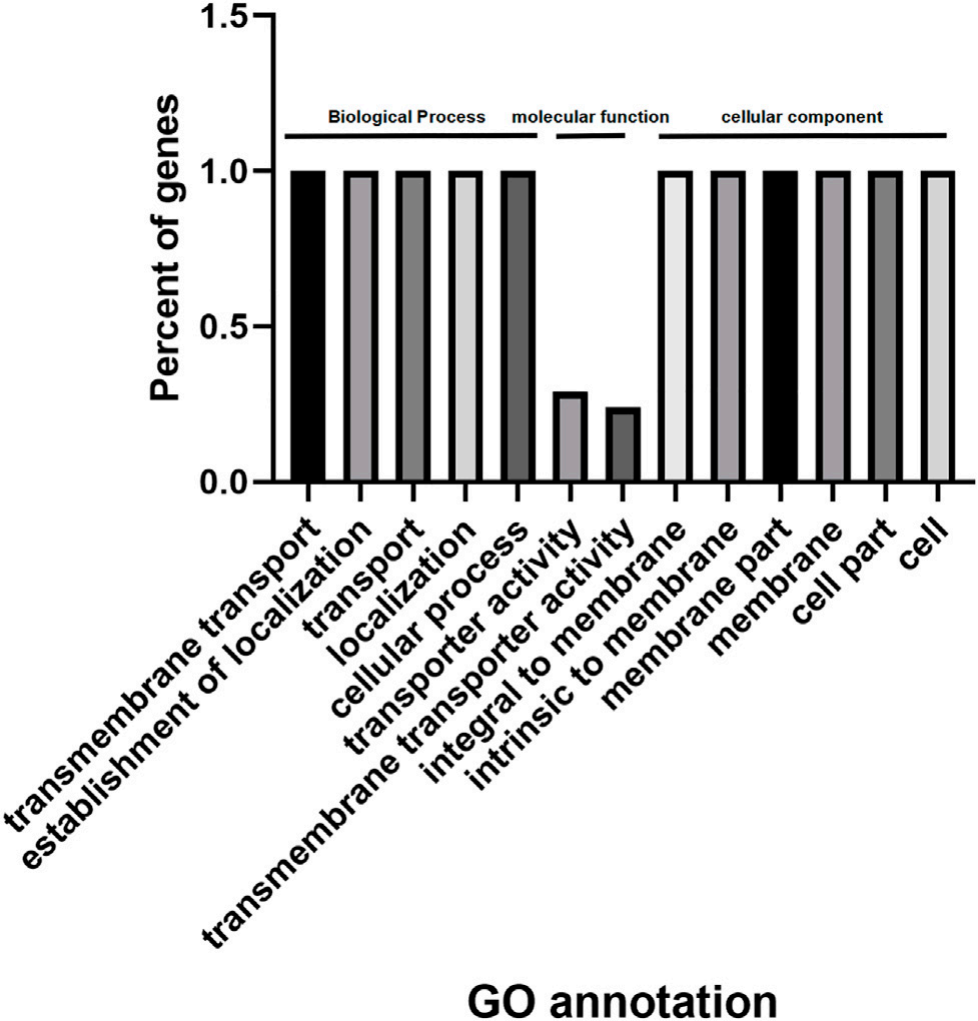
GO analysis of *PdpapMFS* genes.

**FIGURE 14:**
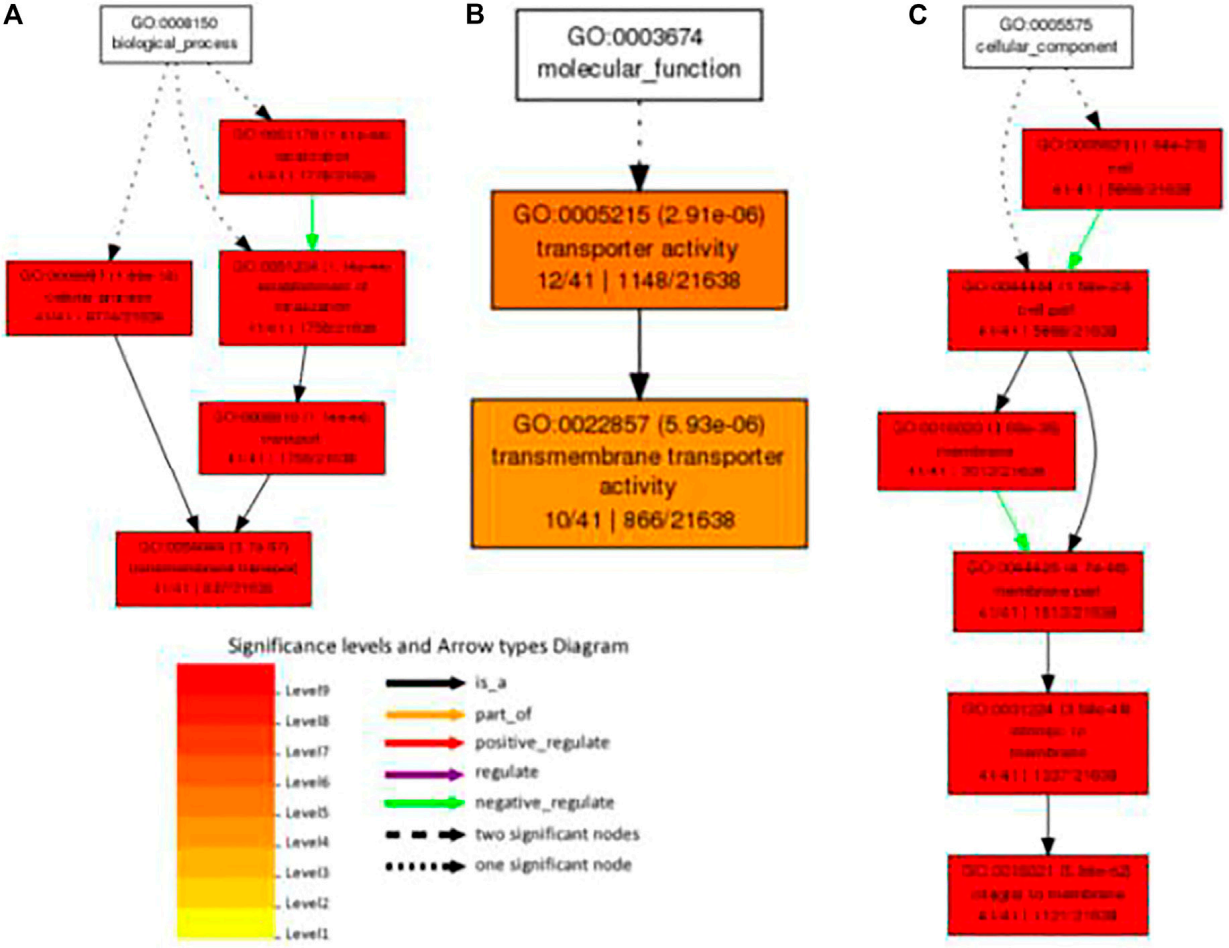
GO analysis of *PdpapMFS* genes in different classifications. **(A)** GO analysis of based on biological processes. **(B)** GO analysis of based on molecular functions. **(C)** GO analysis of based on cellular components.

Using agriGO, we performed gene set enrichment analysis with *PdPapMFS* genes (Figure 13). Based on the annotations, these genes can participate in a variety of biological processes (Figure 14A; Supplementary Table S14), such as in process terms “transmembrane transport,” “establishment of localization,” and “cellular process.” Many *PdPapMFS* genes are enriched in various molecular functions (Figure 14B; Supplementary Table S14), such as in function terms “transporter activity,” and “transmembrane transporter activity.” Regarding cellular localization (Figure 14C; Supplementary Table S14), *PdPapMFS* genes are enriched in localization terms “integral to membrane,” “intrinsic to membrane,” and “cell part.” It is worth mentioning that, many *MFS* genes are enriched in “transmembrane transport,” “transport,” “transporter activity,” “transmembrane transporter activity,” “membrane,” “integral to membrane,” and “intrinsic to membrane.” That also proved our speculation that MFS genes are related to the transmembrane transport function of substances.

## Discussion

The *MFS* gene family exists widely in most organisms and is involved in many critical activities. Some MFS proteins play key roles in immunological processes, such as viral invasion and pathogen resistance. In this study, we identified 41 *PtrMFS* genes in the *P. trichocarpa* genome. The genes were divided into six groups based on amino acid sequence similarity. The largest group was MFS_4, which had nine genes. Analysis of *PtrMFS* protein domains showed that most contain the major facilitator superfamily transporter domain, while others contain a high-affinity nitrate transporter domain and a tetracycline resistance protein domain. We found that many unannotated domains exist in these proteins, which may be responsible for the diverse functions of *PtrMFS*s.

The number of introns varies significantly among the *PtrMFS* gene family. As expected, there is less variability in the structure of introns and exons in genes within the same group. Previous studies have shown that reduction in the number of introns can shorten the time from transcription to translation, thereby promoting rapid gene expression during environmental changes (Jeffares et al., 2008). We found that there are relatively few introns in groups MFS_3, MFS_5, and MFS_6. Overall, 41.46% of the *PtrMFS* genes contain 1–3 introns. Therefore, *PtrMFS* genes may be involved in rapid response to environmental changes.

PtrMFS proteins contained multiple transmembrane domains with the annotated function of material transport. PtrMFS proteins can bind with macromolecular compounds in order to transport them (Wannes and Thomas, 2014). Studies have shown that N-glycosylation sites are essential for protein folding and material transport (Fujii et al., 2006). Pathogen tolerance of poplar may be related to the presence of defensive compounds that function in transporting pathogen toxins out of host cells.

Cis-acting elements contained in promoter regions play key roles in gene regulation and expression. Our analysis of cis-acting elements helped to identify genes with specific functions, such as genes related to stress resistance and plant development. We found that elements related to hormone response and regulation of stresses are present in promoters of almost all *PtrMFS* genes. Thus, the results showed that *PtrMFS* genes may play a key role in regulating responses of poplar to multiple stressors.

Gene families can contain large subfamilies as a result of events such as segmental duplication, tandem duplication, or conversion events (Cannon et al., 2004; Kong et al., 2010). Duplication events can promote the emergence of new genes, which can contribute to increasing the diversity of gene functions, and can effectively improve the ability of plants to adapt to different environments (Flagel and Wendel, 2009). Studies have shown that poplar has undergone at least three rounds of whole genome duplications, in addition to multi-segment duplications, tandem duplications, and transposition events (Tuskan et al., 2006; Wang et al., 2019). In this study, we identified both tandem and segmental duplicates among *PtrMFS* genes. Results showed that segmental duplication events (8 pairs) occurred more frequently than tandem duplication events (3 pairs), suggesting a potentially important role of segmental duplication events in expansion of the *PtrMFS* gene family. Analysis of duplicate gene pairs found that the *Ka/Ks* ratios were all less than 1; therefore, we infer that *PtrMFS* genes have undergone a process of purification and selection during evolutionary processes.

To explore the evolutionary relationships of *MFS* genes among different species, we analyzed the collinearity between *PtrMFS* genes and counterparts from three dicotyledonous and one monocotyledonous plant. Results showed that *PtrMFS* genes have more collinearity with dicotyledonous plants than with monocotyledonous plants. In addition, species with relatively close evolutionary relationships have more collinear gene pairs; we found that *PtrMFS* genes have the most homology with genes from *E. grandis* and the least homology with genes from *O. sativa*.

Analysis of RNA-Seq data showed that poplar *MFS* genes were differentially expressed over time under *F. oxysporum* stress. There were 11 upregulated and 17 downregulated genes shared across all time points after T0. Results revealed that the plants had complex responses in the regulatory networks after different lengths of time post-inoculation. Functional annotations indicated that *PtrMFS* genes play an important role in regulation of material transportation. For example, AT1G08090 (homologous to *PtrMFS24*) encodes a main component of nitrate affinity transport system in roots. Its post-translational regulation mechanism plays a key role in the control of nitrate absorption in roots (Jacquot et al., 2019). AT1G63010 (homologous to *PtrMFS12*) is a tonoplast phosphate transporter, and its ectopic expression can regulate strawberry fruit ripening and quality through sucrose transport (Liu et al., 2015; Huang et al., 2019). AT3G43790 (homologous to *PtrMFS26*) is a functional transporter that mediates K^+^ and Cs^+^ influx when heterologously expressed in yeast (Estelle et al., 2015). AT4G00370 (homologous to *PtrMFS34*) encodes ascorbate transporters on chloroplast envelope membranes, which are necessary for plants to tolerate strong light (Miyaji et al., 2015). Pathogens may activate signal pathways that induce similar cellular responses.

Results of co-expression analysis is useful to find genes with similar expression patterns. These genes are tightly co-regulated and closely related in function. They can also play a role in the same signaling pathway or physiological process (Mathias et al., 2015). We constructed gene co-expression networks to explore the functional relevance of *PtrMFS* genes. *PtrMFS* genes in gene networks are cross-linked, which suggests complex regulation of *PtrMFS* genes in response to *F. oxysporum* stress. We analyzed enrichment of gene sets and found that most genes in the networks are related to transmembrane transport process and membrane part components. Evidence further indicates that poplar *MFS* genes play important roles in the functional regulation of transmembrane transport.

*MFS* genes are known to be involved in the process of material transportation and may play important roles in improving plant resistance to pathogens. In this study, we systematically analyzed properties and expression levels of poplar *MFS* genes. Further study should be conducted to functionally characterize these *MFS* genes. Additionally, a large number of genes related to pathogens resistance need to be mined to realize *F. oxysporum* tolerance of poplars.

## Data Availability

The original contributions presented in the study are included in the article/Supplementary Material, further inquiries can be directed to the corresponding authors.

## References

[B1] Atin S. Rajnikant S. Tapas B. Timsy B. Ranjana P. (2017). Fosfomycin Resistance in *Acinetobacter Baumannii* Is Mediated by Efflux through a Major Facilitator Superfamily (MFS) Transporter—AbaF. J. Antimicrob. Chemoth. 72, 68–74. 10.1093/jac/dkw382 27650185

[B2] Bagchi S. Perland E. Hosseini K. Lundgren J. Al-Walai N. Kheder S. (2020). Probable Role for Major Facilitator Superfamily Domain Containing 6 (MFSD6) in the Brain during Variable Energy Consumption. Int. J. Neurosci. 130, 476–489. 10.1080/00207454.2019.1694020 31906755

[B3] Bailey T. L. Williams N. Misleh C. Li W. W. (2006). MEME: Discovering and Analyzing DNA and Protein Sequence Motifs. Nucleic Acids Res. 34, W369–W373. 10.1093/nar/gkl198 16845028 PMC1538909

[B5] Cannon S. B. Mitra A. Baumgarten A. Young N. D. May G. (2004). The Roles of Segmental and Tandem Gene Duplication in the Evolution of Large Gene Families in *Arabidopsis thaliana* . BMC Plant Biol. 4, 10–768. 10.1186/1471-2229-4-10 15171794 PMC446195

[B6] Chen C. J. Xia R. Chen H. He Y. H. (2018). TBtools, a Toolkit for Biologists Integrating Various HTS-Data Handling Tools with a User-Friendly Interface. Mol. Plant 3, 1–7.

[B7] Chen D. E. Podell S. Sauer J.-D. Swanson M. S. Saier M. H. (2008). The Phagosomal Nutrient Transporter (Pht) Family. Microbiol 154, 42–53. 10.1099/mic.0.2007/010611-0 18174124

[B8] Chen L.-H. Tsai H.-C. Yu P.-L. Chung K.-R. (2017). A Major Facilitator Superfamily Transporter-Mediated Resistance to Oxidative Stress and Fungicides Requires Yap1, Skn7, and MAP Kinases in the Citrus Fungal Pathogen *Alternaria alternata* . Plos One 12, e0169103. 10.1371/journal.pone.0169103 28060864 PMC5218470

[B9] Chen Y. Zhou G. Liu J. (2019). A Major Facilitator Superfamily Transporter inColletotrichum fructicola(CfMfs1) Is Required for Sugar Transport, Appressorial Turgor Pressure, Conidiation and Pathogenicity. For. Path. 49, e12558. 10.1111/efp.12558

[B10] Choi K.-J. Kim H.-B. Park S.-H. (2012). The Comparison of Postoperative Pain: Total Laparoscopic Hysterectomy versus Vaginal Hysterectomy. Korean J. Obstet. Gynecol. 55, 384–391. 10.5468/kjog.2012.55.6.384

[B11] Chun L. I. Sun C. Y. Chen J. Lin Y. P. Wang Y. Zhang M. P. (2018). Research Advances in the Major Facilitator Superfamily. Biotechnol. Bull. 34, 43–49.

[B12] Damian S. Andrea F. Michael K. Milan S. Alexander R. Pablo M. (2011). The STRING Database in 2011: Functional Interaction Networks of Proteins, Globally Integrated and Scored. Nucleic Acids Res. 39, 561–568. 10.1093/nar/gkq1331 PMC3013807 21045058

[B13] de Ramón-Carbonell M. López-Pérez M. González-Candelas L. Sánchez-Torres P. (2019). *PdMFS1* Transporter Contributes to *Penicilliun Digitatum* Fungicide Resistance and Fungal Virulence during Citrus Fruit Infection. JoF 5, 100. 10.3390/jof5040100 PMC6958471 31635246

[B14] Del Sorbo G. Schoonbeek H.-j. De Waard M. A. (2000). Fungal Transporters Involved in Efflux of Natural Toxic Compounds and Fungicides. Fungal Genet. Biol. 30, 1–15. 10.1006/fgbi.2000.1206 10955904

[B15] Diao J. Wang J. Q. Zhang P. Hao X. Wang Y. Liang L. W. (2021). Transcriptome Analysis Reveals the Important Role of *WRKY28* in *Fusarium Oxysporum* Resistance. Front. Plant Sci. 12, 1–17. 10.3389/fpls.2021.720679 PMC8418079 34490017

[B16] dos Santos S. C. Teixeira M. C. Dias P. J. Sã¡-Correia I. (2014). MFS Transporters Required for Multidrug/multixenobiotic (MD/MX) Resistance in the Model Yeast: Understanding Their Physiological Function through post-genomic Approaches. Front. Physiol. 5, 180. 10.3389/fphys.2014.00180 24847282 PMC4021133

[B17] Du Z. Zhou X. Ling Y. Zhang Z. SuAgrigo Z. (2010). agriGO: a GO Analysis Toolkit for the Agricultural Community. Nucleic Acids Res. 38, W64–W70. 10.1093/nar/gkq310 20435677 PMC2896167

[B18] Elbourne L. D. H. Tetu S. G. Hassan K. A. Paulsen I. T. (2017). TransportDB 2.0: a Database for Exploring Membrane Transporters in Sequenced Genomes from All Domains of Life. Nucleic Acids Res. 45, D320–D324. 10.1093/nar/gkw1068 27899676 PMC5210551

[B20] Finn R. D. Bateman A. Clements J. Coggill P. Eberhardt R. Y. Eddy S. R. (2014). Pfam: the Protein Families Database. Nucl. Acids Res. 42, D222–D230. 10.1093/nar/gkt1223 24288371 PMC3965110

[B21] Flagel L. E. Wendel J. F. (2009). Gene Duplication and Evolutionary novelty in Plants. New Phytol. 183, 557–564. 10.1111/j.1469-8137.2009.02923.x 19555435

[B22] Fluman N. Ryan C. M. Whitelegge J. P. Bibi E. (2012). Dissection of Mechanistic Principles of a Secondary Multidrug eFflux Protein. Mol. Cel 47, 777–787. 10.1016/j.molcel.2012.06.018 PMC5524571 22841484

[B23] Fujii Y. Khoshnoodi J. Takenaka H. Hosoyamada M. Nakajo A. Bessho F. (2006). The Effect of Dexamethasone on Defective Nephrin Transport Caused by ER Stress: A Potential Mechanism for the Therapeutic Action of Glucocorticoids in the Acquired Glomerular Diseases. Kidney Int. 69, 1350–1359. 10.1038/sj.ki.5000317 16531978

[B24] Geourjon C. Deléage G. (1995). SOPMA: Significant Improvements in Protein Secondary Structure Prediction by Consensus Prediction from Multiple Alignments. Bioinformatics 11, 681–684. 10.1093/bioinformatics/11.6.681 8808585

[B65] Gasteiger E. Gattiker A. Hoogland C. Ivanyi I. Appel R. D. Bairoch A. (2003). ExPASy: The Proteomics Server for In-Depth Protein Knowledge and Analysis. Nucleic Acids Res. 31, 3784–3788. 10.1093/nar/gkg563 12824418 PMC168970

[B25] Goodstein D. M. Shu S. Howson R. Neupane R. Hayes R. D. Fazo J. (2012). Phytozome: a Comparative Platform for green Plant Genomics. Nucleic Acids Res. 40, D1178–D1186. 10.1093/nar/gkr944 22110026 PMC3245001

[B26] Grossman T. H. (2016). Tetracycline Antibiotics and Resistance. Cold Spring Harb Perspect. Med. 6, a025387. 10.1101/cshperspect.a025387 26989065 PMC4817740

[B27] Hayashi K. Schoonbeek H.-j. De Waard M. A. (2002). Bcmfs1 , a Novel Major Facilitator Superfamily Transporter from Botrytis Cinerea , Provides Tolerance towards the Natural Toxic Compounds Camptothecin and Cercosporin and towards Fungicides. Appl. Environ. Microbiol. 68, 4996–5004. 10.1128/aem.68.10.4996-5004.2002 12324349 PMC126426

[B28] Huang Y.-C. Chang Y.-L. Hsu J.-J. Chuang H.-w. (2008). Transcriptome Analysis of Auxin-Regulated Genes of *Arabidopsis thaliana* . Gene 420, 118–124. 10.1016/j.gene.2008.05.017 18577427

[B29] Huang Y. Xu P. H. Hou B. Z. Shen Y. Y. (2019). Strawberry Tonoplast Transporter, FaVPT1, Controls Phosphate Accumulation and Fruit Quality. Plant Cel Environ 42, 2715–2729. 10.1111/pce.13598 31151133

[B30] Ideker T. (2011). Cytoscape 2.8: New Features for Data Integration and Network Visualization. Bioinformatics 27, 431–432. 21149340 10.1093/bioinformatics/btq675 PMC3031041

[B52] Ivica L. Peer B. (2018). 20 Years of the SMART Protein Domain Annotation Resource. Nucleic Acids Res. 46, D493–D496. 10.1093/nar/gkx922 29040681 PMC5753352

[B31] Jacquot A. Chaput V. Mauries A. Li Z. Lejay L. (2019). NRT2.1 Phosphorylation Prevents Root High Affinity Nitrate Uptake Activity in *Arabidopsis thaliana* . Bioinformatics 228, 1038–432. 10.1101/583542 32463943

[B32] Jaina M. Finn R. D. Eddy S. R. Alex B. Marco P. (2013). Challenges in Homology Search: HMMER3 and Convergent Evolution of Coiled-Coil Regions. Nucleic Acids Res. 41, e121. 23598997 10.1093/nar/gkt263 PMC3695513

[B33] Jeffares D. C. Penkett C. J. Bähler J. (2008). Rapidly Regulated Genes Are Intron Poor. Trends Genet. 24, 375–378. 10.1016/j.tig.2008.05.006 18586348

[B81] Kang P. Z. Bai X. J. Zhang L. R. Du Y. N. Zhang Z. K. (2018). Biological Characteristics of *Fusarium oxysporium* (Schl.) f.sp.*Cucumerinum Owen* . Nor. Horticul. 22, 65–69.

[B34] Kenneth J. L. Thomas D. S. (2002). Analysis of Relative Gene Expression Data Using Real-Time Quantitative PCR and the 2^-ΔΔCT^ Method. Methods 25, 402–408. 10.1006/meth.2001.1262 11846609

[B35] Kong H. Landherr L. L. Frohlich M. W. Leebens-Mack J. Ma H. dePamphilis C. W. (2010). Patterns of Gene Duplication in the Plant SKP1 Gene Family in Angiosperms: Evidence for Multiple Mechanisms of Rapid Gene Birth. Plant J. 50, 873–885. 10.1111/j.1365-313X.2007.03097.x 17470057

[B4] Kumar K. (2014). SPSS for Applied Sciences: Basic Statistical Testing. J. R. Stat. Soc. A. Stat. 177, 990–990. 10.1111/rssa.12082_2

[B36] Lee J. (2015). MFS Transporter Superfamily: Modelling and Dynamics. Oxford: Doctoral, University of Oxford.

[B37] Lin H.-C. Yu P.-L. Chen L.-H. Tsai H.-C. Chung K.-R. (2018). A Major Facilitator Superfamily Transporter Regulated by the Stress-Responsive Transcription Factor Yap1 Is Required for Resistance to Fungicides, Xenobiotics, and Oxidants and Full Virulence in *Alternaria alternata* . Front. Microbiol. 9, 2229. 10.3389/fmicb.2018.02229 30279684 PMC6153361

[B38] Liu J. Yang L. Luan M. Wang Y. Zhang C. Zhang B. (2015). A Vacuolar Phosphate Transporter Essential for Phosphate Homeostasis in *Arabidopsis* . Proc. Natl. Acad. Sci. U S A. 112, E6571–E6578. 10.1073/pnas.1514598112 26554016 PMC4664319

[B39] Lorca G. L. Barabote R. D. Zlotopolski V. Tran C. Winnen B. Hvorup R. N. (2007). Transport Capabilities of Eleven Gram-Positive Bacteria: Comparative Genomic Analyses. Biochim. Biophys. Acta (Bba) - Biomembranes 1768, 1342–1366. 10.1016/j.bbamem.2007.02.007 17490609 PMC2592090

[B40] Lu L. Giang H. Loussaert D. F. Wang H. (2010). Functional Expression of Higher Plant Nitrate Transporters in Pichia Pastoris.

[B41] Marcet-Houben M. Ballester A.-R. de la Fuente B. Harries E. Marcos J. F. González-Candelas L. (2012). Genome Sequence of the Necrotrophic Fungus *Penicillium digitatum*, the Main Postharvest Pathogen of Citrus. BMC Genomics 13, 646. 10.1186/1471-2164-13-646 23171342 PMC3532085

[B42] Marger M. D. Saier M. H. (1993). A Major Superfamily of Transmembrane Facilitators that Catalyse Uniport, Symport and Antiport. Trends Biochem. Sci. 18, 13–20. 10.1016/0968-0004(93)90081-w 8438231

[B43] Maria N. G. Esther N. U. Dale N. R. Pedro M. B. Paula D. (2019). More Transporters, More Substrates: The *Arabidopsis* Major Facilitator Superfamily Revisited. Mol. Plant 12, 1182–1202. 31330327 10.1016/j.molp.2019.07.003

[B44] Martin B. Hiba S. Michael S. Alona R. Magnus K. Benjamin A. H. (2021). Ferulic Acid, an Abundant maize Phenolic, Regulates ABC and MFS Transporter Gene Expression in the maize Pathogen *Cochliobolus Heterostrophus* . J. Plant Dis. Protect. 128, 1383–1391.

[B45] Mathias C. Miin-Huey L. Huey-Jiunn B. Kuang R. (2007). Deletion of a MFS Transporter-like Gene in *Cercospora Nicotianae* Reduces Cercosporin Toxin Accumulation and Fungal Virulence. Febs Lett. 581, 489–494. 17250832 10.1016/j.febslet.2007.01.011

[B46] Matthias K. Michaela L. Andreas M. Anne-Sophie W. Sabine F. (2009). Fungicide-driven Evolution and Molecular Basis of Multidrug Resistance in Field Populations of the Grey Mould Fungus *Botrytis Cinerea* . Plos Pathog. 5, e1000696. 20019793 10.1371/journal.ppat.1000696 PMC2785876

[B82] Mathias T. J. Natarajan K. Shukla S. Doshi K. A. Singh Z. N. Ambudkar S. V. (2015). The FLT3 and PDGFR Inhibitor Crenolanib is a Substrate of the Multidrug Resistance Protein ABCB1 but does not Inhibit Transport Function at Pharmacologically Relevant Concentrations. Invest. New Drug. 33, 300–309. 10.1007/s10637-015-0205-y PMC7709138 25597754

[B47] Miyaji T. Kuromori T. Takeuchi Y. Yamaji N. Yokosho K. Shimazawa A. (2015). AtPHT4;4 Is a Chloroplast-Localized Ascorbate Transporter in *Arabidopsis* . Nat. Commun. 6, 5928. 10.1038/ncomms6928 25557369 PMC4308718

[B48] Monalessa F. P. Carolina M. A. S. Elza F. A. Marisa V. Q. Denise M. S. B. (2013). Beginning to Understand the Role of Sugar Carriers in *Colletotrichum Lindemuthianum*: the Function of the Gene *Mfs1* . J. Microbiol. 51, 70–81. 23456714 10.1007/s12275-013-2393-5

[B49] Nicolas M. Jean-Luc B. Naomi T. Marc S. (2005). HTLV-1 Tropism and Envelope Receptor. Oncogene 24, 6016–6025. 16155608 10.1038/sj.onc.1208972

[B50] Omrane S. Audéon C. Ignace A. Duplaix C. Aouini L. Kema G. (2017). Plasticity of the *MFS1* Promoter Leads to Multidrug Resistance in the Wheat Pathogen *Zymoseptoria Tritici* . Msphere 2, 393–417. 10.1128/mSphere.00393-17 PMC5656749 29085913

[B51] Omrane S. Sghyer H. Audéon C. Lanen C. Duplaix C. Walker A.-S. (2015). Fungicide Efflux and the MgMFS1 Transporter Contribute to the Multidrug Resistance Phenotype inZymoseptoria Triticifield Isolates. Environ. Microbiol. 17, 2805–2823. 10.1111/1462-2920.12781 25627815

[B53] Paul H. Keun-Joon P. Takeshi O. Naoya F. Hajime H. Adams-Collier C. J. (2007). WoLF PSORT: Protein Localization Predictor. Nucleic Acids Res. 35, 585–587. 10.1093/nar/gkl929 PMC1933216 17517783

[B54] Paulsen I. T. Brown M. H. Skurray R. A. (1996). Proton-dependent Multidrug Efflux Systems. Microbiol. Rev. 60, 575–608. 10.1128/mr.60.4.575-608.1996 8987357 PMC239457

[B55] Quevillon E. Silventoinen V. Pillai S. Harte N. Mulder N. Apweiler R. (2005). InterProScan: Protein Domains Identifier. Nucleic Acids Res. 33, W116–W120. 10.1093/nar/gki442 15980438 PMC1160203

[B56] Ramin R. Maarten A. D. W. Gert H. J. K. Lute-Harm Z. (2007). MgMfs1, a Major Facilitator Superfamily Transporter from the Fungal Wheat Pathogen *Mycosphaerella Graminicola*, Is a strong Protectant against Natural Toxic Compounds and Fungicides. Fungal Genet. Biol. 44, 378–388. 17107817 10.1016/j.fgb.2006.09.007

[B57] Ramón-Carbonell M. d. Sánchez-Torres P. (2017). Involvement of *Penicillium digitatum PdSUT1* in Fungicide Sensitivity and Virulence during Citrus Fruit Infection. Microbiol. Res. 203, 57–67. 10.1016/j.micres.2017.06.008 28754208

[B58] Reisz J. A. Bechtold E. King S. B. Poole L. B. Furdui C. M. (2013). Thiol-blocking Electrophiles Interfere with Labeling and Detection of Protein Sulfenic Acids. Febs J. 280, 6150–6161. 10.1111/febs.12535 24103186 PMC3928805

[B19] Remy E. Cabrito T. R. Batista R. A. Teixeira M. C. Sá-Correia I. Duque P. (2015). The Major Facilitator Superfamily Transporter ZIFL2 Modulates Cesium and Potassium Homeostasis in *Arabidopsis* . Plant Cel Physiol 56, 148–162. 10.1093/pcp/pcu157 25378686

[B59] Rombauts S. Dehais P. Van Montagu M. Rouze P. (1999). PlantCARE, a Plant Cis-Acting Regulatory Element Database. Nucleic Acids Res. 27, 295–296. 10.1093/nar/27.1.295 9847207 PMC148162

[B60] Saier M. H. Paulsen I. T. (2011). Phylogeny of Multidrug Transporters. Semin. Cel Dev. Biol. 12, 205–213. 10.1006/scdb.2000.0246 11428913

[B61] Saier M. H. Reddy V. S. Tamang D. G. Åke V. (2009). The Transporter Classification Database: Recent Advances. Nucleic Acids Res. 44, 372–379. 10.1093/nar/gkn862 PMC2686586 19022853

[B62] Setyowati T. U. Carissa I. I. Anom B. Takashi Y. Chen X. Y. Kyoko N. (2020). Identification and Functional Characterization of *Penicillium marneffei* Major Facilitator Superfamily (MFS) Transporters. Biosci. Biotech. Bioch. 84, 1373–1383. 10.1080/09168451.2020.1732185 32163007

[B63] Tamura K. Peterson D. Peterson N. Stecher G. Nei M. Kumar S. (2011). MEGA5: Molecular Evolutionary Genetics Analysis Using Maximum. Mol. Biol. Evol. 28, 2731–2739. 10.1093/molbev/msr121 21546353 PMC3203626

[B64] Tang H. Bowers J. E. Wang X. Ming R. Alam M. Paterson A. H. (2008). Synteny and Collinearity in Plant Genomes. Science 320, 486–488. 10.1126/science.1153917 18436778

[B66] Torsten S. Jürgen K. Nicolas G. Peitsch M. C. (2003). SWISS-MODEL: an Automated Protein Homology-Modeling Server. Nucleic Acids Res. 31, 3381–3385. 10.1126/science.1128691 12824332 PMC168927

[B67] Tuskan G. A. Difazio S. Jansson S. Bohlmann J. Grigoriev I. Hellsten U. (2006). The Genome of Black Cottonwood, Populus trichocarpa (Torr. & Gray). Science 313, 1596–1604. 10.1126/science.1128691 16973872

[B68] Ulrich O. Ahrens C. H. Sebastian M. Bernd W. (2014). Protter: Interactive Protein Feature Visualization and Integration with Experimental Proteomic Data. Bioinformatics 30, 884–886. 10.1093/bioinformatics/btt658 24162465

[B69] Utami S. T. Indriani C. I. Bowolaksono A. Yaguchi T. Chen X. Niimi K. (2020). Identification and Functional Characterization of *Penicillium marneffei* Major Facilitator Superfamily (MFS) Transporters. Biosci. Biotechnol. Biochem. 84, 1373–1383. 10.1080/09168451.2020.1732185 32163007

[B70] Vela-Corcía D. Aditya Srivastava D. Dafa-Berger A. Rotem N. Barda O. Levy M. (2019). MFS Transporter from *Botrytis Cinerea* Provides Tolerance to Glucosinolate-Breakdown Products and Is Required for Pathogenicity. Nat. Commun. 10, 2886. 10.1038/s41467-019-10860-3 31253809 PMC6599007

[B71] Wang D.-M. Liu L. Fan L. Zou Z.-J. Zhang L.-N. Yang S. (2012). Expression Level of DEK in Chronic Lymphocytic Leukemia Is Regulated by Fludarabine and Nutlin-3 Depending on P53 Status. Cancer Biol. Ther. 13, 1522–1528. 10.4161/cbt.22252 23052131 PMC3542244

[B72] Wang J. Y. Sun X. P. Lin L. Y. Zhang T. Y. Li H. Y. (2012). PdMfs1, a Major Facilitator Superfamily Transporter from *Penicillium digitatum*, Is Partially Involved in the Imazalil-Resistance and Pathogenicity. Afr. J. Microbiol. Res. 6, 95–105. 10.5897/ajmr11.1045

[B73] Wang Y. Salasini B. C. Khan M. Devi B. Bush M. Subramaniam R. (2019). Clade I TGACG-Motif Binding Basic Leucine Zipper Transcription Factors Mediate BLADE-ON-PETIOLE-dependent Regulation of Development. Plant Physiol. 180, 937–951. 10.1104/pp.18.00805 30923069 PMC6548253

[B74] Wang Y. Tang H. Debarry J. D. Tan X. Li J. Wang X. (2012). MCScanX: a Toolkit for Detection and Evolutionary Analysis of Gene Synteny and Collinearity. Nucleic Acids Res. 40, e49. 10.1093/nar/gkr1293 22217600 PMC3326336

[B75] Wannes D. Thomas V. L. (2014). The ABC Gene Family in Arthropods: Comparative Genomics and Role Ininsecticide Transport and Resistance. Insect Biochem. Mol. 45, 89–110. 10.1016/j.ibmb.2013.11.001 24291285

[B76] Wu Z. Wang S. Yuan Y. Zhang T. Liu J. Liu D. (2016). A Novel Major Facilitator Superfamily Transporter in *Penicillium digitatum* (PdMFS2) Is Required for Prochloraz Resistance, Conidiation and Full Virulence. Biotechnol. Lett. 38, 1349–1357. 10.1007/s10529-016-2113-4 27146209

[B77] Yen M. R. Chen J. S. Marquez J. L. Sun E. I. Saier M. H. (2010). Multidrug Resistance: Phylogenetic Characterization of Superfamilies of Secondary Carriers that Include Drug Exporters. Methods Mol. Biol. 637, 47–64. 10.1007/978-1-60761-700-6_3 20419429

[B78] Zhai Z. H. Chen X. N. Wang J. (2008). Primer Design with Primer Premier 5.0. Northwest. Med. Educ. 4, 695–698.

[B79] Zhang T. Cao Q. Li N. Liu D. Yuan Y. (2020). Transcriptome Analysis of Fungicide-Responsive Gene Expression Profiles in Two *Penicillium italicum* Strains with Different Response to the Sterol Demethylation Inhibitor (DMI) Fungicide Prochloraz. BMC Genomics 21, 156. 10.1186/s12864-020-6564-6 32050894 PMC7017498

[B80] Zhang Z. Li J. Zhao X.-Q. Wang J. Wong G. K.-S. Yu J. (2006). KaKs_Calculator: Calculating Ka and Ks through Model Selection and Model Averaging. Genomics, Proteomics & Bioinformatics 4, 259–263. 10.1016/s1672-0229(07)60007-2 PMC5054075 17531802

