# Supplementary material for: Genome-Wide Analysis of Major Facilitator Superfamily and Its Expression in Response of Poplar to Fusarium oxysporum
Source: Front Genet. 2021 Oct 22;12:769888. doi: 10.3389/fgene.2021.769888 (PMC8567078; doi:10.3389/fgene.2021.769888)
Supplement: Supplementary file 1 [file DataSheet2.PDF]

Table S4. Annotations of PtrMFS protein sequence motifs

Name	Logo	Sequence	Description of Pfam	Distribution in PtrMFSs
Motif 1	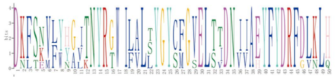	DKFSNVFYHGIKNYRGWILALSYGYCFGVELTIDNIVAEYFYDRFDLKLH	LytB protein	PtrMFS7; PtrMFS24; PtrMFS25; PtrMFS32; PtrMFS36; PtrMFS37
Motif 2	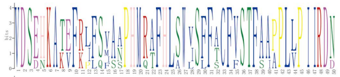	VDSEHKATEFRLFSVAAPHMRAFHLSWVSFFACFVSTFAAPPLLPIIRDN	High-Affinity Nitrate Transporter; Major Facilitator Superfamily	PtrMFS7; PtrMFS24; PtrMFS25; PtrMFS32; PtrMFS36; PtrMFS37
Motif 3	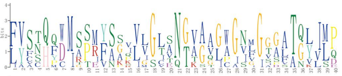	FVSTQFWMSSMFSARVVGTANGVAAGWGNLGGGATQLIMP	High-Affinity Nitrate Transporter; Major Facilitator Superfamily	PtrMFS5; PtrMFS7; PtrMFS8; PtrMFS14; PtrMFS16; PtrMFS24; PtrMFS25; PtrMFS27; PtrMFS30; PtrMFS32; PtrMFS34; PtrMFS36; PtrMFS37; PtrMFS41;
Motif 4	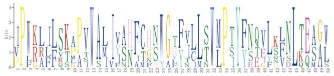	IPWKLILSKAPVWALIISHFCHNWGTFILLTWMPTYYNQVLKFNLTESGL	Major Facilitator Superfamily	PtrMFS4; PtrMFS5; PtrMFS16; PtrMFS27; PtrMFS30; PtrMFS34; PtrMFS39; PtrMFS41;
Motif 5	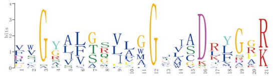	FWGYALSQLPGGWLAKIFGGR	Major Facilitator Superfamily	PtrMFS2; PtrMFS3; PtrMFS4; PtrMFS5; PtrMFS6; PtrMFS7; PtrMFS8; PtrMFS10; PtrMFS11; PtrMFS14; PtrMFS15; PtrMFS16; PtrMFS17; PtrMFS18; PtrMFS19; PtrMFS20; PtrMFS21; PtrMFS24; PtrMFS25; PtrMFS26; PtrMFS27; PtrMFS28; PtrMFS30; PtrMFS32; PtrMFS34; PtrMFS36; PtrMFS37; PtrMFS38; PtrMFS39; PtrMFS40; PtrMFS41;
Motif 6	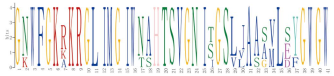	GNWFGKRKRGLIMGIWNAHTSVGNITGSLIAASVLEYGWGW	Major Facilitator Superfamily	PtrMFS2; PtrMFS3; PtrMFS10; PtrMFS11; PtrMFS40;
Motif 7	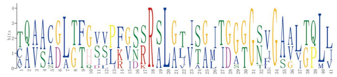	TAVAADLGTQDLIKGNSRALATVSAIIDGTGSVGAAVGPLL	Major Facilitator Superfamily Transporter	PtrMFS2; PtrMFS3; PtrMFS7; PtrMFS10; PtrMFS11; PtrMFS24; PtrMFS25; PtrMFS32; PtrMFS36; PtrMFS37; PtrMFS40;
Motif 8	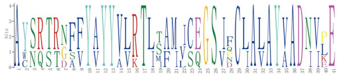	AYSRTTNFFYAYYVLRTLTAMICEGSINCLALAYVADNVLE	Major Facilitator Superfamily; Tetracycline Resistance Protein, Class A-Like	PtrMFS8; PtrMFS13; PtrMFS14; PtrMFS22; PtrMFS29; PtrMFS31;
Motif 9	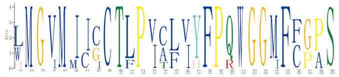	LMGVMIICCTLPICFIHFPQWGGMFCGPS	none	PtrMFS7; PtrMFS24; PtrMFS25; PtrMFS32; PtrMFS36; PtrMFS37;

Motif 10	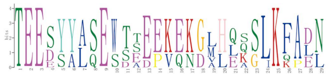	TEESYYASEWSEEEKQKGMHQQSLKFAEN	none	PtrMFS1; PtrMFS12; PtrMFS24; PtrMFS25; PtrMFS32; PtrMFS36; PtrMFS37;
Motif 11	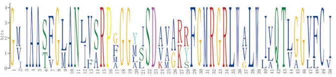	GMIAASFGLANIVSRPGGGMISDAVAKRFGMRGRLWALWIVQTLGGVFCI	High-Affinity Nitrate Transporter	PtrMFS7; PtrMFS24; PtrMFS25; PtrMFS32; PtrMFS36; PtrMFS37;
Motif 12	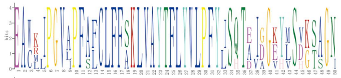	EAWKIPGVAPFALCLFFAKLVAYTFLYWLPFYISQTAIDGKYLSDGTAGN	Major Facilitator Superfamily;	PtrMFS2; PtrMFS3; PtrMFS10; PtrMFS11; PtrMFS40
Motif 13	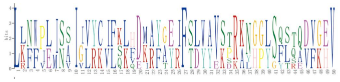	LKNWPLISSIIVYCVFQLHDMAYAEIFSLWAVSPRKNGGLSFSTADVGEV	Major Facilitator Superfamily; Protein Zinc Induced Facilitator	PtrMFS1; PtrMFS12; PtrMFS15; PtrMFS21; PtrMFS26; PtrMFS33; PtrMFS38;
Motif 14	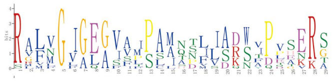	RAFMGIGEGVAMPAMNNILSKWIPVSERS	Major Facilitator Superfamily	PtrMFS4; PtrMFS5; PtrMFS6; PtrMFS16; PtrMFS18; PtrMFS19; PtrMFS20; PtrMFS27; PtrMFS28; PtrMFS30; PtrMFS34; PtrMFS39; PtrMFS41;
Motif 15	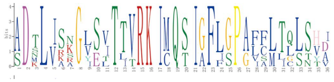	ADTLVSKGLSVTTVRKIMQSIGFLGPAFFLTQLSHI	Anion Transporter; Solute Carrier Family	PtrMFS4; PtrMFS5; PtrMFS16; PtrMFS27; PtrMFS34; PtrMFS39; PtrMFS41;
Motif 16	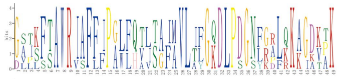	GAIKFTAWRIAFFIPALFQTLSAFAVLIFGKDLPDGNFRRLQKAGDKTK	High-Affinity Nitrate Transporter	PtrMFS7; PtrMFS24; PtrMFS25; PtrMFS32; PtrMFS36; PtrMFS37;
Motif 17	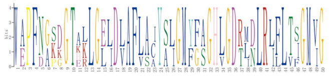	WEPFNGKDGTEKLGLIDVAFLACYSLGMFGAGHLGDTLDLRLFLTSGMIG	Major Facilitator Superfamily Transporter	PtrMFS2; PtrMFS3; PtrMFS10; PtrMFS11; PtrMFS40
Motif 18	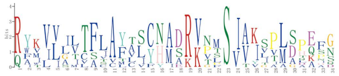	RYMVLLVTFIAYTSYHASRKPSSIVKSALDPEPN	none	PtrMFS2; PtrMFS3; PtrMFS4; PtrMFS10; PtrMFS11; PtrMFS16; PtrMFS27; PtrMFS30; PtrMFS34; PtrMFS39; PtrMFS40; PtrMFS41;
Motif 19	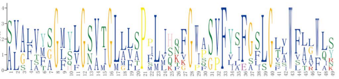	SLAFVYSGMYLGSVTGLAFSPFLIHQFGWPSVFYSFGSLGTVWFAAWLS	Major Facilitator Superfamily	PtrMFS4; PtrMFS5; PtrMFS16; PtrMFS27; PtrMFS30; PtrMFS34; PtrMFS39; PtrMFS41;
Motif 20	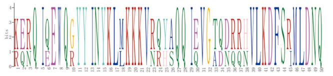	KERQIQEWQGYYINYKLMKKKVRQYAQQIEVGTQDRRHVLKDFSRMLDNQ	SPX domain	PtrMFS1; PtrMFS12; PtrMFS33;
