# Supplementary material for: Genome-Wide Analysis of Major Facilitator Superfamily and Its Expression in Response of Poplar to Fusarium oxysporum
Source: Front Genet. 2021 Oct 22;12:769888. doi: 10.3389/fgene.2021.769888 (PMC8567078; doi:10.3389/fgene.2021.769888)
Supplement: Supplementary file 2 [file DataSheet4.PDF]

Table S7. The result of the topological heterogeneity model prediction

Gene name	Name	Locus tag	Number of transmembrane helical segments	Number of N-glycosylation sites	Signal peptides	Topological heterogeneity model
LOC7478080	PtrMFS1	POPTR_001G111400v3	11	3	None	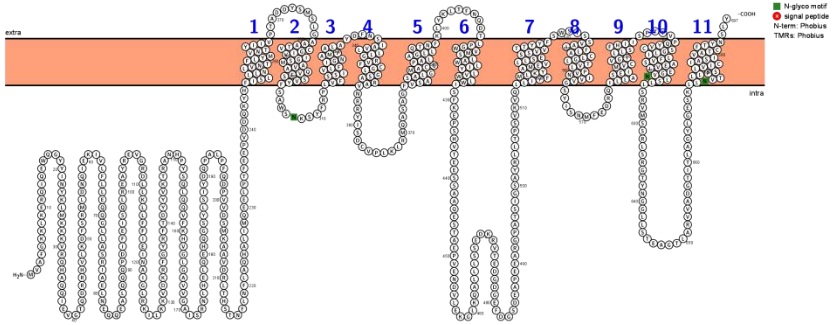
LOC18094006	PtrMFS2	POPTR_001G124200v3	12	3	None	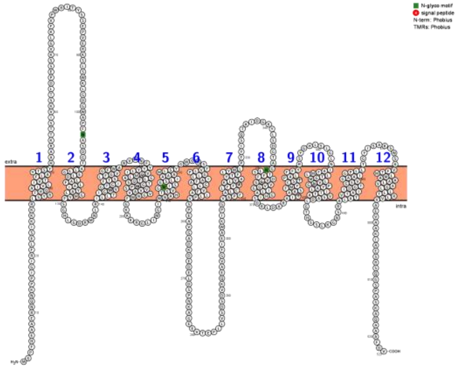

LOC18094568	PtrMFS3	POPTR_001G152300v3	12	4	None	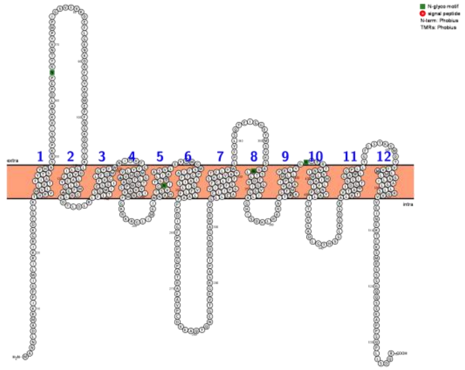
LOC7468011	PtrMFS4	POPTR_001G248200v3	12	4	None	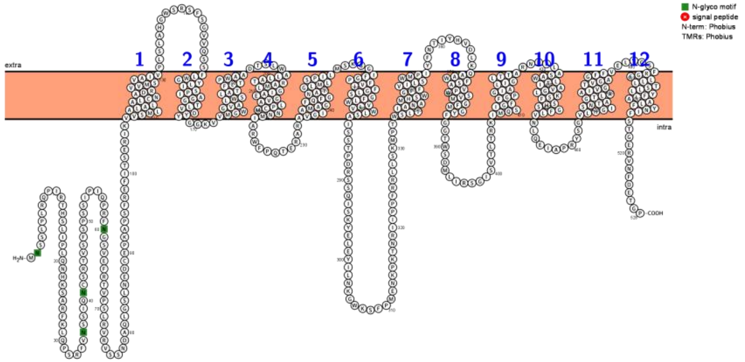

LOC7478542	PtrMFS5	POPTR_001G249800v3	10	2	None	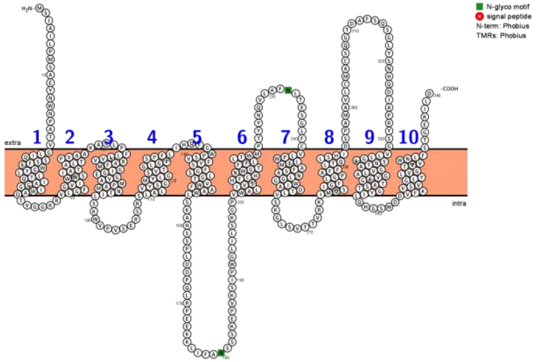
LOC7470780	PtrMFS6	POPTR_001G286600v3	11	4	None	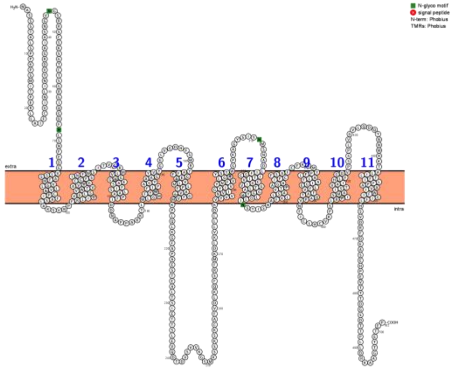

LOC7487860	PtrMFS7	POPTR_001G348300v3	11	2	None	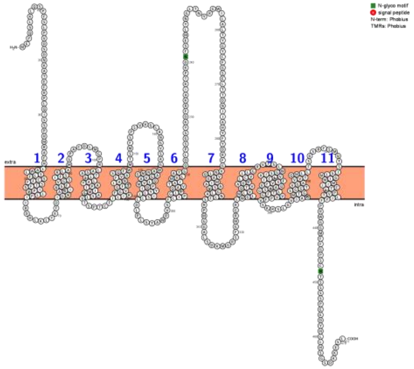
LOC7496897	PtrMFS8	POPTR_002G016200v3	11	2	Yes	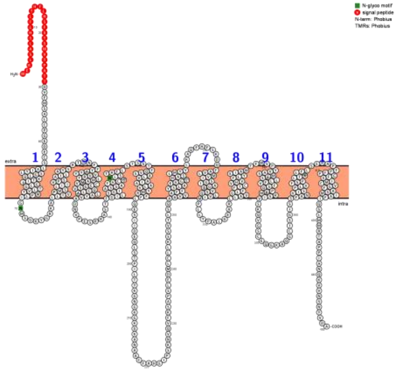

LOC18096251	PtrMFS9	POPTR_002G106900v3	11	1	None	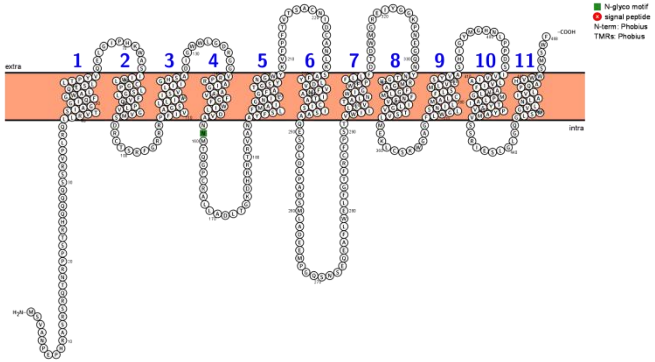
LOC18096883	PtrMFS10	POPTR_003G082400v3	12	2	None	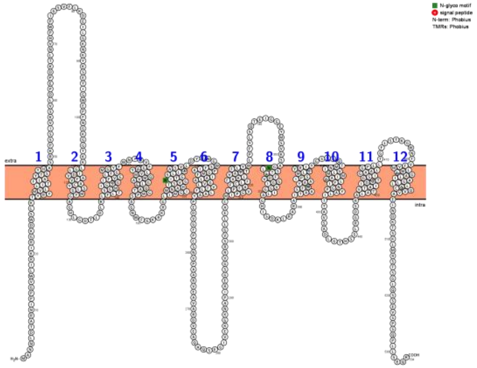

LOC7465506	PtrMFS11	POPTR_003G109300v3	12	2	None	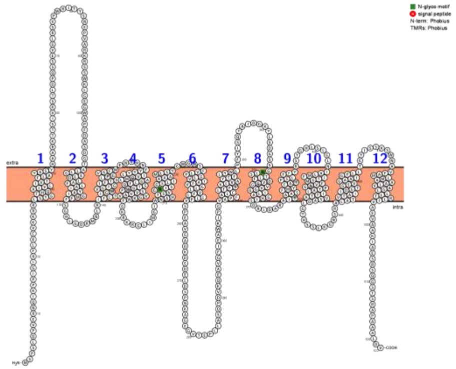
LOC7479251	PtrMFS12	POPTR_003G120600v3	11	3	None	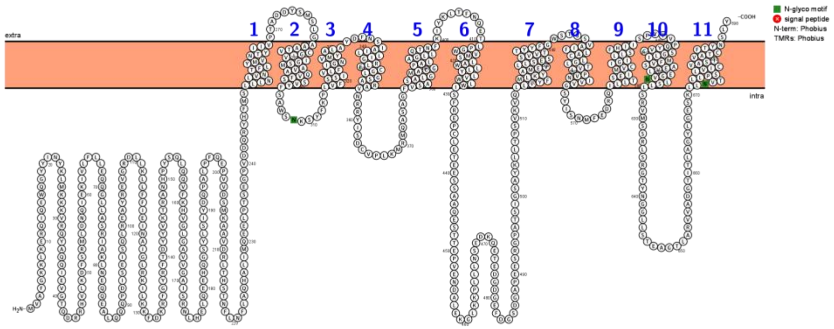
LOC7453664	PtrMFS13	POPTR_004G178600v3	9	1	None	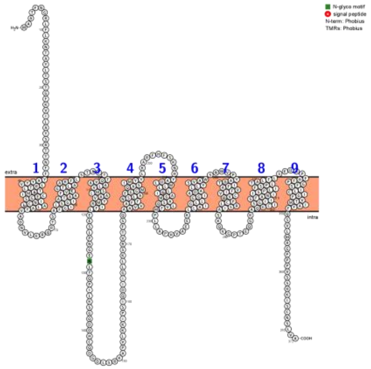

LOC7494001	PtrMFS14	POPTR_005G245900v3	11	3	Yes	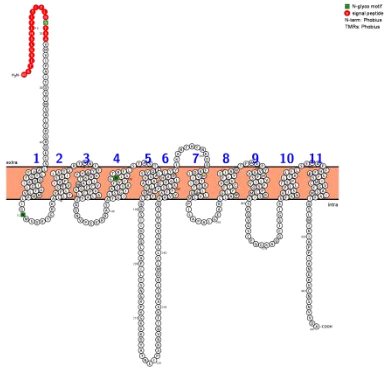
LOC7479627	PtrMFS15	POPTR_006G026200v3	11	3	None	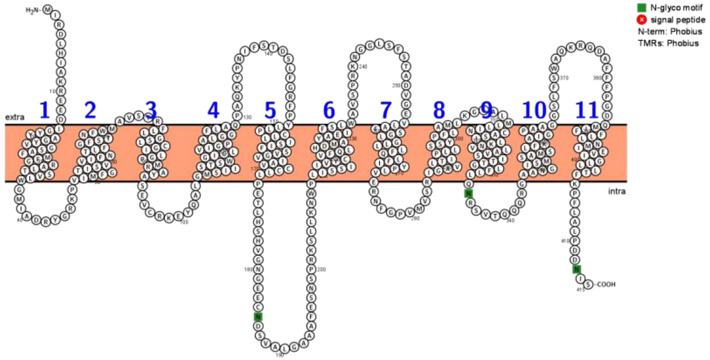

LOC18099899	PtrMFS16	POPTR_006G062300v3	10	9	None	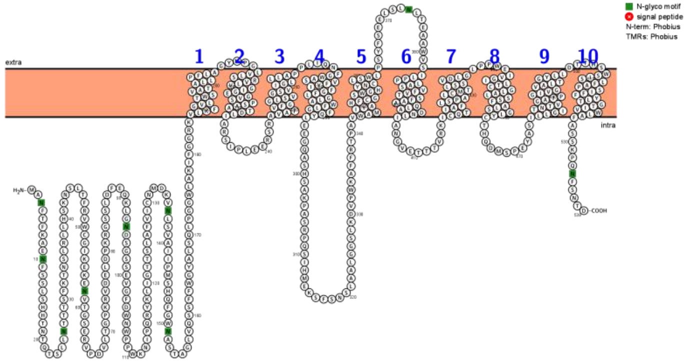
LOC7497754	PtrMFS17	POPTR_007G003100v3	12	3	None	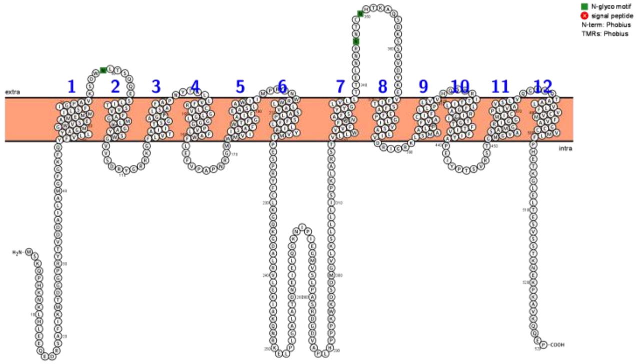

LOC7473146	PtrMFS18	POPTR_007G030800v3	11	2	Yes	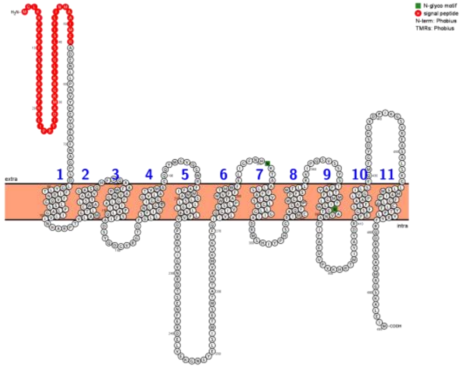
LOC18100946	PtrMFS19	POPTR_007G091700v3	7	0	Yes	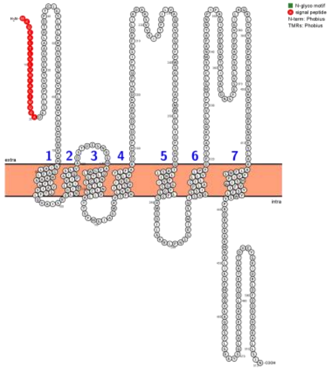

LOC7490218	PtrMFS20	POPTR_007G091800v3	7	2	None	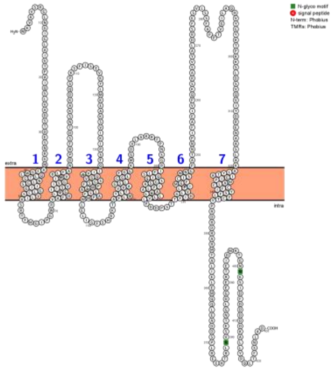
LOC7486851	PtrMFS21	POPTR_008G010600v3	9	3	None	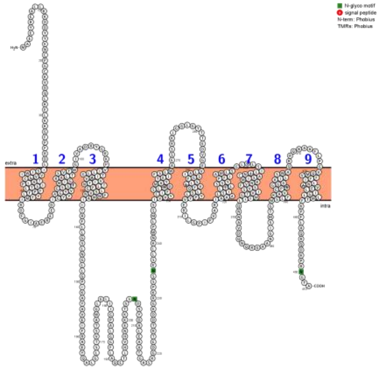

LOC7486914	PtrMFS22	POPTR_008G022100v3	9	1	None	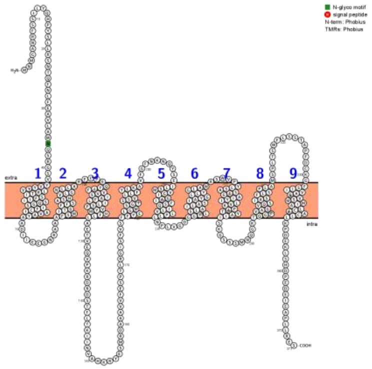
LOC7463316	PtrMFS23	POPTR_009G006400v3	13	0	None	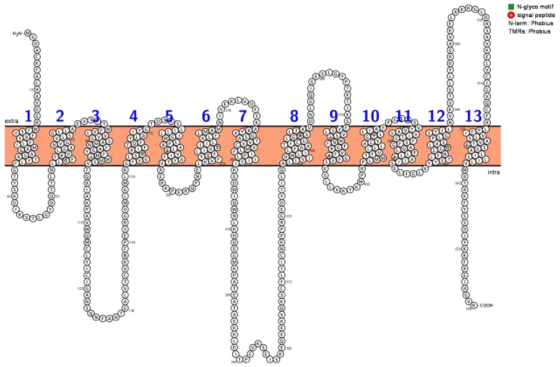

LOC7463308	PtrMFS24	POPTR_009G008500v3	12	2	None	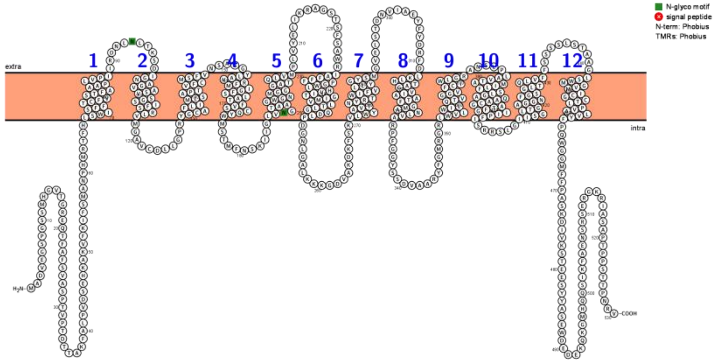
LOC7463307	PtrMFS25	POPTR_009G008600v3	12	2	None	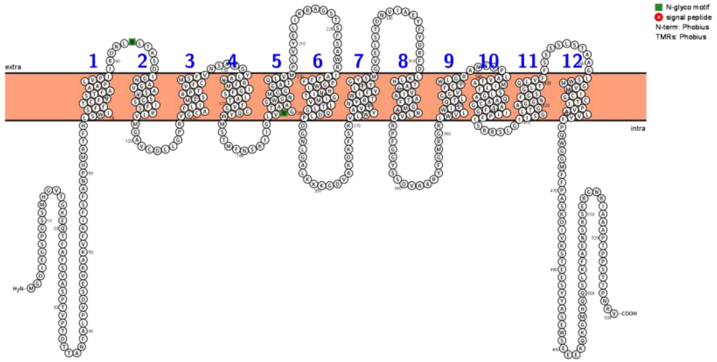

LOC7488173	PtrMFS26	POPTR_009G021700v3	11	0	None	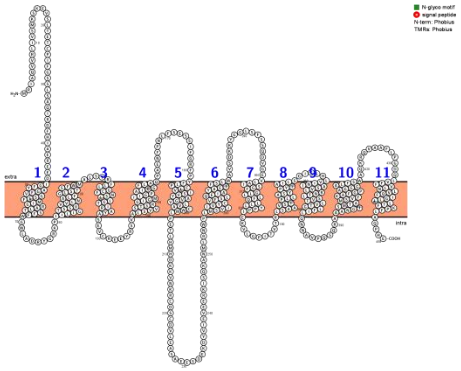
LOC7481487	PtrMFS27	POPTR_009G043800v3	11	3	None	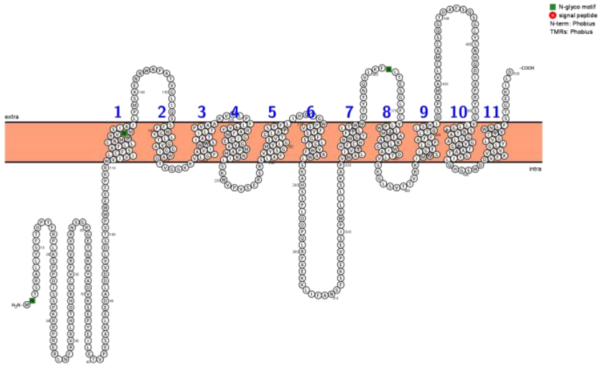

LOC7478769	PtrMFS28	POPTR_009G081100v3	9	2	Yes	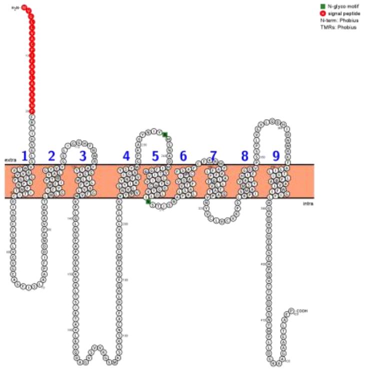
LOC7474984	PtrMFS29	POPTR_009G138900v3	10	2	None	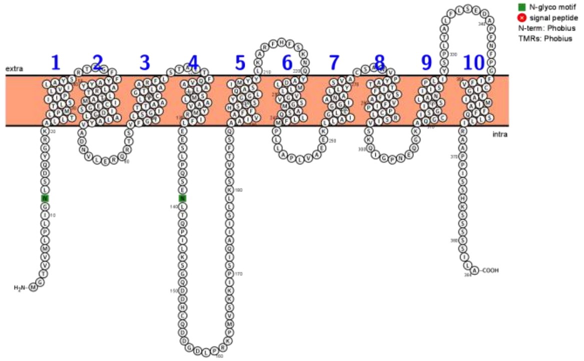

LOC7463360	PtrMFS30	POPTR_009G168200v3	11	2	None	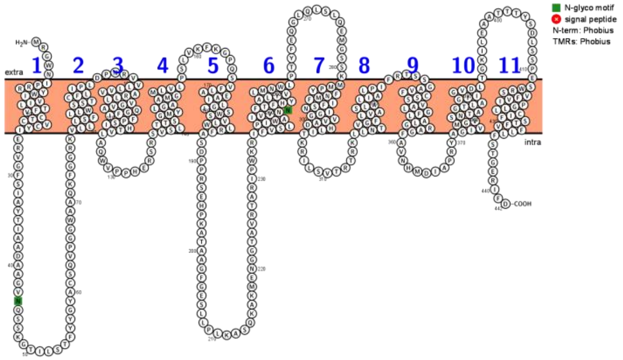
LOC7477174	PtrMFS31	POPTR_010G237300v3	10	2	None	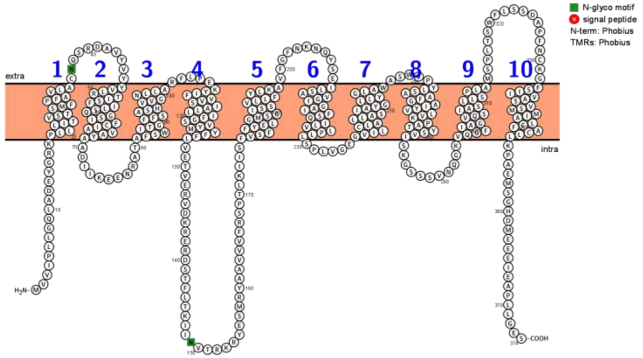

LOC7458066	PtrMFS32	POPTR_012G087700v3	11	1	None	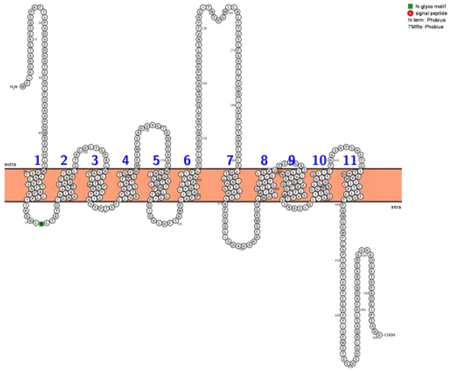
LOC7496936	PtrMFS33	POPTR_014G078000v3	11	5	None	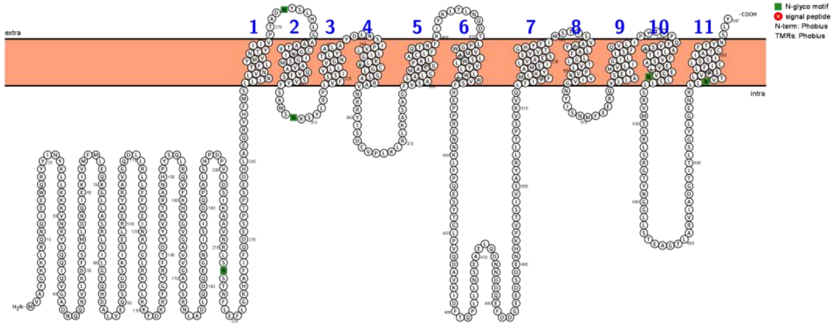
LOC7496975	PtrMFS34	POPTR_014G085700v3	11	2	None	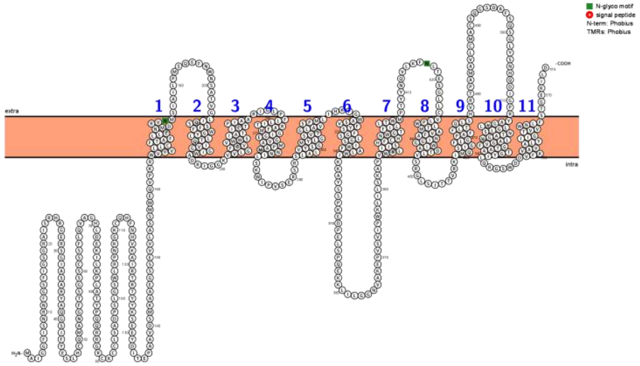

LOC7462526	PtrMFS35	POPTR_015G067000v3	14	4	None	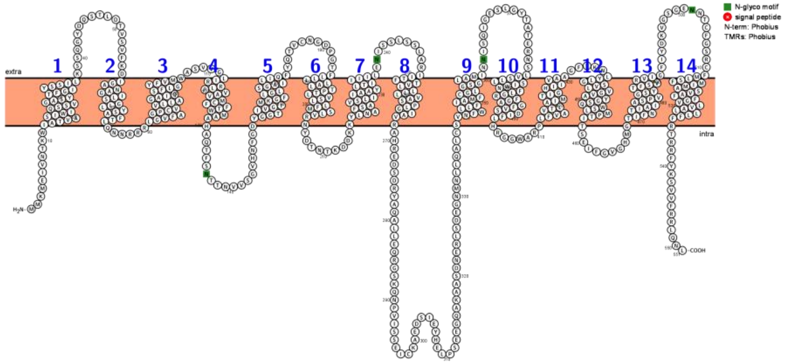
LOC7457762	PtrMFS36	POPTR_015G081300v3	11	1	None	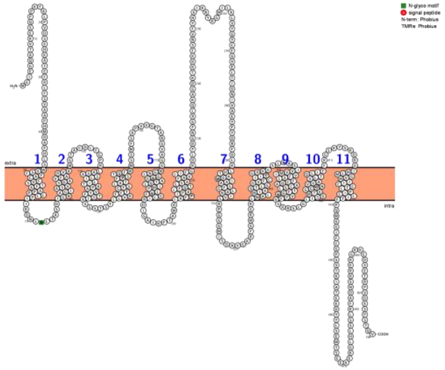

LOC7457764	PtrMFS37	POPTR_015G081500v3	11	1	None	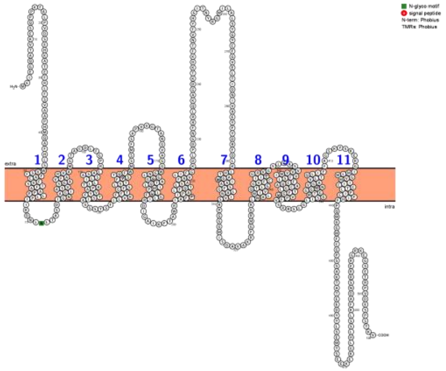
LOC7455882	PtrMFS38	POPTR_016G024400v3	10	4	None	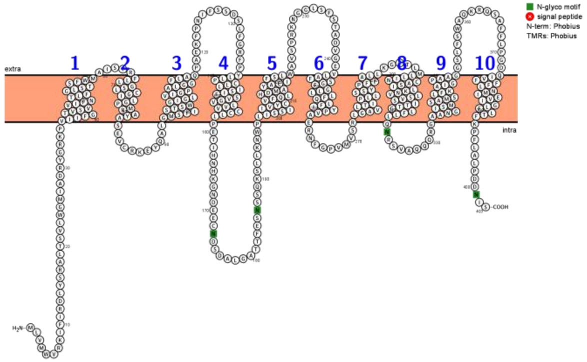

LOC7466045	PtrMFS39	POPTR_016G111000v3	12	3	None	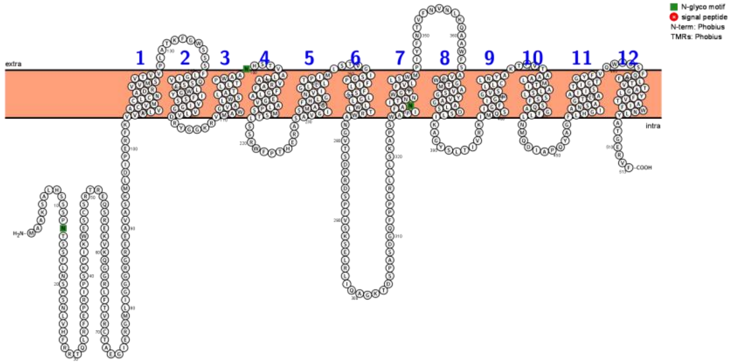
LOC7489481	PtrMFS40	POPTR_018G115000v3	11	5	None	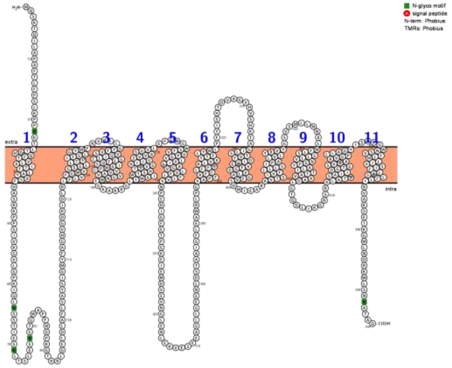

LOC18111057	PtrMFS41	POPTR_018G121600v3	10	6	None	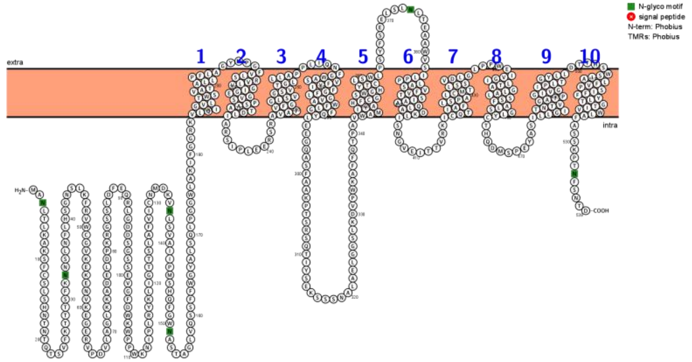
