# Supplementary material for: Genome-Wide Analysis of Major Facilitator Superfamily and Its Expression in Response of Poplar to Fusarium oxysporum
Source: Front Genet. 2021 Oct 22;12:769888. doi: 10.3389/fgene.2021.769888 (PMC8567078; doi:10.3389/fgene.2021.769888)
Supplement: Supplementary file 4 [file Table8.DOCX]

**Table S11**. The homologous relationships between *PtrMFS* genes and other species

| ***Populus trichocarpa* - *Eucalyptus* *grandis*** | | ***Populus trichocarpa* - *Solanum lycopersicum*** | | ***Populus trichocarpa* - *Arabidopsis thaliana*** | | ***Populus trichocarpa* - *Oryza sativa*** | |
| --- | --- | --- | --- | --- | --- | --- | --- |
| Potri.001G111400 | Eucgr.E00526 | Potri.003G120600 | Solyc08g007800 | Potri.003G120600 | AT4G22990 | Potri.001G111400 | LOC_Os04g48390 |
| Potri.003G120600 | Eucgr.E00526 | Potri.004G178600 | Solyc10g055770 | Potri.007G030800 | AT4G36790 | Potri.001G152300 | LOC_Os02g43620 |
| Potri.004G178600 | Eucgr.I01458 | Potri.005G245900 | Solyc04g081990 | Potri.015G067000 | AT1G18940 | Potri.001G248200 | LOC_Os01g63290 |
| Potri.007G030800 | Eucgr.I02474 | Potri.009G008600 | Solyc06g074990 | Potri.001G111400 | AT4G22990 | Potri.003G082400 | LOC_Os02g43620 |
| Potri.008G022100 | Eucgr.J00999 | Potri.002G016200 | Solyc04g081990 | Potri.001G152300 | AT4G17550 | Potri.008G022100 | LOC_Os11g05390 |
| Potri.010G237300 | Eucgr.J00999 | Potri.001G111400 | Solyc08g007800 | Potri.001G249800 | AT2G29650 | Potri.016G111000 | LOC_Os05g37820 |
| Potri.001G111400 | Eucgr.D01930 | Potri.001G111400 | Solyc08g080200 | Potri.001G348300 | AT5G14570 | Potri.018G115000 | LOC_Os06g08170 |
| Potri.001G152300 | Eucgr.E00368 | Potri.001G152300 | Solyc06g060910 | Potri.002G106900 | AT1G09960 |  |  |
| Potri.001G248200 | Eucgr.J01945 | Potri.002G016200 | Solyc12g094400 | Potri.003G082400 | AT4G17550 |  |  |
| Potri.001G249800 | Eucgr.J01933 | Potri.002G106900 | Solyc04g076960 | Potri.003G120600 | AT1G63010 |  |  |
| Potri.002G016200 | Eucgr.F01701 | Potri.003G120600 | Solyc08g080200 | Potri.004G178600 | AT2G16970 |  |  |
| Potri.002G106900 | Eucgr.F00464 | Potri.004G178600 | Solyc01g111840 | Potri.006G062300 | AT5G20380 |  |  |
| Potri.003G082400 | Eucgr.E00368 | Potri.005G245900 | Solyc12g094400 | Potri.007G030800 | AT2G18590 |  |  |
| Potri.003G120600 | Eucgr.D01930 | Potri.006G062300 | Solyc07g007560 | Potri.007G091800 | AT5G10190 |  |  |
| Potri.004G178600 | Eucgr.F01701 | Potri.007G030800 | Solyc02g085670 | Potri.009G006400 | AT2G28120 |  |  |
| Potri.005G245900 | Eucgr.F01701 | Potri.007G091800 | Solyc04g055080 | Potri.009G008600 | AT5G60770 |  |  |
| Potri.006G026200 | Eucgr.K01705 | Potri.008G022100 | Solyc11g007130 | Potri.009G043800 | AT2G29650 |  |  |
| Potri.006G062300 | Eucgr.C00489 | Potri.009G008600 | Solyc11g069750 | Potri.009G138900 | AT2G16970 |  |  |
| Potri.007G030800 | Eucgr.H01238 | Potri.009G081100 | Solyc01g106420 | Potri.014G085700 | AT4G00370 |  |  |
| Potri.007G091800 | Eucgr.H04799 | Potri.010G237300 | Solyc11g007130 | Potri.015G067000 | AT1G74780 |  |  |
| Potri.008G022100 | Eucgr.G02698 | Potri.014G078000 | Solyc08g080200 | Potri.016G111000 | AT2G38060 |  |  |
| Potri.009G006400 | Eucgr.B03936 | Potri.014G085700 | Solyc01g094720 | Potri.018G115000 | AT2G13100 |  |  |
| Potri.009G008600 | Eucgr.J00898 | Potri.015G067000 | Solyc03g113340 | Potri.018G121600 | AT5G20380 |  |  |
| Potri.009G043800 | Eucgr.J01933 | Potri.016G111000 | Solyc09g013150 |  |  |  |  |
| Potri.009G138900 | Eucgr.I01458 | Potri.018G115000 | Solyc12g010440 |  |  |  |  |
| Potri.009G168200 | Eucgr.I01531 | Potri.018G121600 | Solyc07g007560 |  |  |  |  |
| Potri.010G237300 | Eucgr.G02698 |  |  |  |  |  |  |
| Potri.014G085700 | Eucgr.D02302 |  |  |  |  |  |  |
| Potri.015G067000 | Eucgr.K02903 |  |  |  |  |  |  |
| Potri.016G111000 | Eucgr.B02783 |  |  |  |  |  |  |
| Potri.018G115000 | Eucgr.C04092 |  |  |  |  |  |  |
| Potri.018G121600 | Eucgr.C00489 |  |  |  |  |  |  |
