# Supplementary material for: Genome-Wide Analysis of Major Facilitator Superfamily and Its Expression in Response of Poplar to Fusarium oxysporum
Source: Front Genet. 2021 Oct 22;12:769888. doi: 10.3389/fgene.2021.769888 (PMC8567078; doi:10.3389/fgene.2021.769888)
Supplement: Supplementary file 7 [file Table7.DOCX]

**Table S10**. The list of 8 pairs repetitive events in *PtrMFS* genes and its Ka/Ks ratio.

| **Gene-1** | **Locus tag** | **Gene-2** | **Locus tag** | **Ka** | **Ks** | **Ka/Ks** |
| --- | --- | --- | --- | --- | --- | --- |
| *PtrMFS11* | POPTR_003G109300v3 | *PtrMFS2* | POPTR_001G124200v3 | 0.048521363 | 0.20550671 | 0.236105979 |
| *PtrMFS10* | POPTR_003G082400v3 | *PtrMFS3* | POPTR_001G152300v3 | 0.055935003 | 0.168377378 | 0.332200227 |
| *PtrMFS5* | POPTR_001G249800v3 | *PtrMFS27* | POPTR_009G043800v3 | 0.032135197 | 0.188105641 | 0.170835903 |
| *PtrMFS6* | POPTR_001G286600v3 | *PtrMFS28* | POPTR_009G081100v3 | 0.064753726 | 0.181105988 | 0.357546025 |
| *PtrMFS8* | POPTR_002G016200v3 | *PtrMFS13* | POPTR_004G178600v3 | 0.240120906 | 1.676698943 | 0.143210507 |
| *PtrMFS14* | POPTR_005G245900v3 | *PtrMFS8* | POPTR_002G016200v3 | 0.050296738 | 0.295283564 | 0.170333687 |
| *PtrMFS13* | POPTR_004G178600v3 | *PtrMFS29* | POPTR_009G138900v3 | 0.081052596 | 0.21686483 | 0.373747076 |
| *PtrMFS38* | POPTR_016G024400v3 | *PtrMFS15* | POPTR_006G026200v3 | 0.044899995 | 0.281296441 | 0.15961807 |
