# Supplementary material for: Genome-Wide Analysis of Major Facilitator Superfamily and Its Expression in Response of Poplar to Fusarium oxysporum
Source: Front Genet. 2021 Oct 22;12:769888. doi: 10.3389/fgene.2021.769888 (PMC8567078; doi:10.3389/fgene.2021.769888)
Supplement: Supplementary file 8 [file DataSheet3.PDF]

**Table S6**. Predition of the protein structure homology-modelling in PtrMFSs

| Name in this paper | Locus tag | Gene Name | Template | Sequence Identity | Description | Homology-modelling |
|---|---|---|---|---|---|---|
| PtrMFS1 | POPTR_001G111400v3 | LOC7478080 | 4w6v.1.A | 12.53% | Di-/tripeptide transporter; Crystal structure of a peptide transporter from Yersinia enterocolitica at 3 A resolution. | 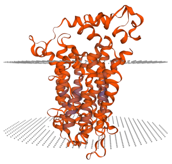 |
| PtrMFS2 | POPTR_001G124200v3 | LOC18094006 | 1pw4.1.A | 20.15% | Glycerol-3-phosphate transporter; Crystal Structure of the Glycerol-3-Phosphate Transporter from E.Coli. | 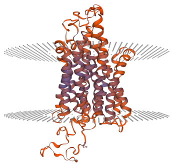 |
| PtrMFS3 | POPTR_001G152300v3 | LOC18094568 | 1pw4.1.A | 20.83% | Glycerol-3-phosphate transporter; Crystal Structure of the Glycerol-3-Phosphate Transporter from E.Coli. | 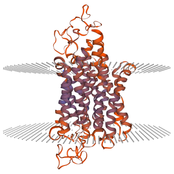 |
| PtrMFS4 | POPTR_001G248200v3 | LOC7468011 | 6v4d.1.A | 29.18% | Vesicular glutamate transporter 2; Structure of the rat vesicular glutamate transporter 2 determined by single particle Cryo-EM. | 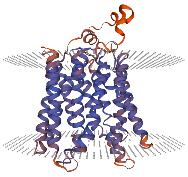 |
| PtrMFS5 | POPTR_001G249800v3 | LOC7478542 | 6v4d.1.A | 37.43% | Vesicular glutamate transporter 2; Structure of the rat vesicular glutamate transporter 2 determined by single particle Cryo-EM. | 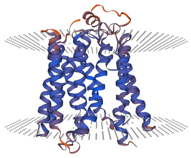 |
| PtrMFS6 | POPTR_001G286600v3 | LOC7470780 | 6e9c.1.A | 18.21% | Major facilitator family transporter; Selenomethionine Derivative Structure of A Bacterial Homolog to Human Lysosomal Transporter, Spinster. | 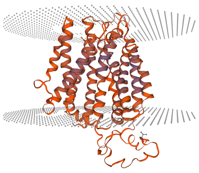 |

| | | | | | | |
|---|---|---|---|---|---|---|
| PtrMFS7 | POPTR_001G348300v3 | LOC7487860 | 6g9x.1.A | 15.71% | Major facilitator superfamily MFS_1; Crystal structure of a MFS transporter at 2.54 Angstroem resolution. | 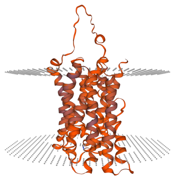 |
| PtrMFS8 | POPTR_002G016200v3 | LOC7496897 | 6s4m.1.A | 19.26% | Major facilitator superfamily domain-containing protein 10; Crystal structure of the human organic anion transporter MFSD10 (TETRAN). | 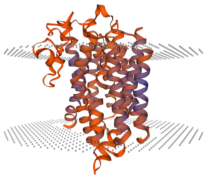 |
| PtrMFS9 | POPTR_002G106900v3 | LOC18096251 | 6gs1.1.A | 15.34% | Dipeptide and tripeptide permease A; Crystal structure of peptide transporter DtpA-nanobody in MES buffer. | 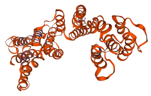 |
| PtrMFS10 | POPTR_003G082400v3 | LOC18096883 | 1pw4.1.A | 20.83% | Glycerol-3-phosphate transporter; Crystal Structure of the Glycerol-3-Phosphate Transporter from E.Coli. | 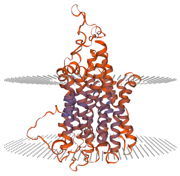 |
| PtrMFS11 | POPTR_003G109300v3 | LOC7465506 | 1pw4.1.A | 20.15% | Glycerol-3-phosphate transporter; Crystal Structure of the Glycerol-3-Phosphate Transporter from E.Coli. | 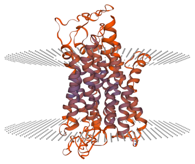 |
| PtrMFS12 | POPTR_003G120600v3 | LOC7479251 | 6s4m.1.A | 17.50% | Major facilitator superfamily domain-containing protein 10. Crystal structure of the human organic anion transporter MFSD10 (TETRAN). | 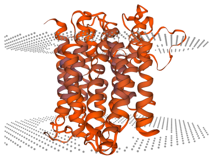 |
| PtrMFS13 | POPTR_004G178600v3 | LOC7453664 | 6s4m.1.A | 18.69% | Major facilitator superfamily domain-containing protein 10. Crystal structure of the human organic anion transporter MFSD10 (TETRAN). | 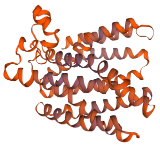 |

| | | | | | | |
|---|---|---|---|---|---|---|
| PtrMFS14 | POPTR_005G245900v3 | LOC7494001 | 6s4m.1.A | 17.66% | Major facilitator superfamily domain-containing protein 10. Crystal structure of the human organic anion transporter MFSD10 (TETRAN). | 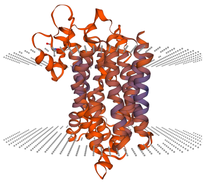 |
| PtrMFS15 | POPTR_006G026200v3 | LOC7479627 | 6s4m.1.A | 20.29% | Major facilitator superfamily domain-containing protein 10. Crystal structure of the human organic anion transporter MFSD10 (TETRAN). | 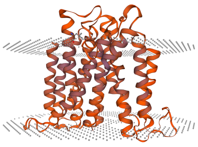 |
| PtrMFS16 | POPTR_006G062300v3 | LOC18099899 | 6v4d.1.A | 29.30% | Vesicular glutamate transporter 2. Structure of the rat vesicular glutamate transporter 2 determined by single particle Cryo-EM. | 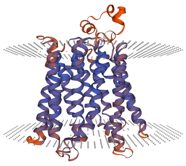 |
| PtrMFS17 | POPTR_007G003100v3 | LOC7497754 | 4j05.1.A | 20.26% | Phosphate transporter. Crystal structure of a eukaryotic phosphate transporter. | 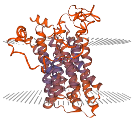 |
| PtrMFS18 | POPTR_007G030800v3 | LOC7473146 | 6eba.1.A | 16.07% | Major facilitator family transporter. Crystal Structure of A Bacterial Homolog to Human Lysosomal Transporter, Spinster, in Inward-facing And Unoccupied Conformation. | 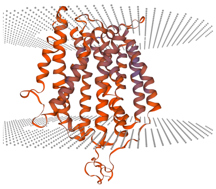 |
| PtrMFS19 | POPTR_007G091700v3 | LOC18100946 | 6e9c.1.A | 18.98% | Major facilitator family transporter. Selenomethionine Derivative Structure of A Bacterial Homolog to Human Lysosomal Transporter, Spinster. | 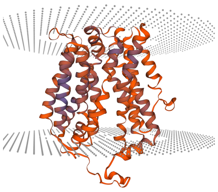 |
| PtrMFS20 | POPTR_007G091800v3 | LOC7490218 | 6e9c.1.A | 18.21% | Major facilitator family transporter. Selenomethionine Derivative Structure of A Bacterial Homolog to Human Lysosomal Transporter, Spinster. | 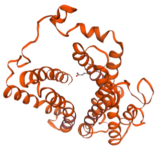 |

| | | | | | | |
|---|---|---|---|---|---|---|
| PtrMFS21 | POPTR_008G010600v3 | LOC7486851 | 6s4m.1.A | 17.71% | Major facilitator superfamily domain-containing protein 10. Crystal structure of the human organic anion transporter MFSD10 (TETRAN). | 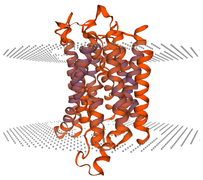 |
| PtrMFS22 | POPTR_008G022100v3 | LOC7486914 | 6s4m.1.A | 18.35% | Major facilitator superfamily domain-containing protein 10. Crystal structure of the human organic anion transporter MFSD10 (TETRAN). | 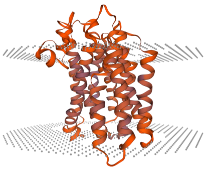 |
| PtrMFS23 | POPTR_009G006400v3 | LOC7463316 | 7ckr.1.A | 14.25% | Monocarboxylate transporter 1. Cryo-EM structure of the human MCT1/Basigin-2 complex in the presence of anti-cancer drug candidate BAY-8002 in the outward-open conformation. | 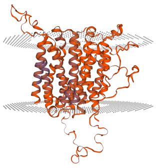 |
| PtrMFS24 | POPTR_009G008500v3 | LOC7463308 | 6g9x.1.A | 16.01% | Major facilitator superfamily MFS_1. Crystal structure of a MFS transporter at 2.54 Angstroem resolution. | 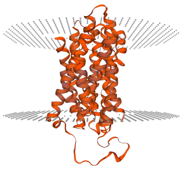 |
| PtrMFS25 | POPTR_009G008600v3 | LOC7463307 | 6g9x.1.A | 16.25% | Major facilitator superfamily MFS_1. Crystal structure of a MFS transporter at 2.54 Angstroem resolution. | 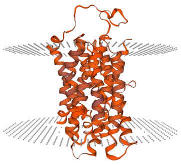 |
| PtrMFS26 | POPTR_009G021700v3 | LOC7488173 | 6s4m.1.A | 15.86% | Major facilitator superfamily domain-containing protein 10. Crystal structure of the human organic anion transporter MFSD10 (TETRAN). | 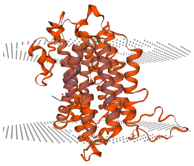 |
| PtrMFS27 | POPTR_009G043800v3 | LOC7481487 | 6v4d.1.A | 37.71% | Vesicular glutamate transporter 2. Structure of the rat vesicular glutamate transporter 2 determined by single particle Cryo-EM. | 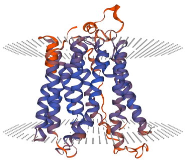 |

| | | | | | | |
|---|---|---|---|---|---|---|
| PtrMFS28 | POPTR_009G081100v3 | LOC7478769 | 7ckr.1.A | 15.45% | Monocarboxylate transporter 1. Cryo-EM structure of the human MCT1/Basigin-2 complex in the presence of anti-cancer drug candidate BAY-8002 in the outward-open conformation. | 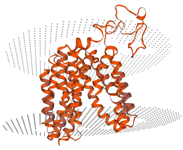 |
| PtrMFS29 | POPTR_009G138900v3 | LOC7474984 | 6s4m.1.A | 21.74% | Major facilitator superfamily domain-containing protein 10. Crystal structure of the human organic anion transporter MFSD10 (TETRAN). | 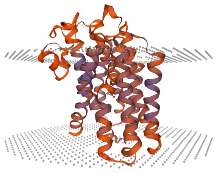 |
| PtrMFS30 | POPTR_009G168200v3 | LOC7463360 | 6v4d.1.A | 31.10% | Vesicular glutamate transporter 2. Structure of the rat vesicular glutamate transporter 2 determined by single particle Cryo-EM. | 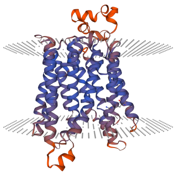 |
| PtrMFS31 | POPTR_010G237300v3 | LOC7477174 | 6s4m.1.A | 21.34% | Major facilitator superfamily domain-containing protein 10. Crystal structure of the human organic anion transporter MFSD10 (TETRAN). | 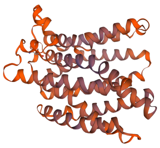 |
| PtrMFS32 | POPTR_012G087700v3 | LOC7458066 | 1pw4.1.A | 18.06% | Glycerol-3-phosphate transporter. Crystal Structure of the Glycerol-3-Phosphate Transporter from E.Coli. | 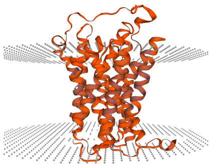 |
| PtrMFS33 | POPTR_014G078000v3 | LOC7496936 | 6s4m.1.A | 17.68% | Major facilitator superfamily domain-containing protein 10. Crystal structure of the human organic anion transporter MFSD10 (TETRAN). | 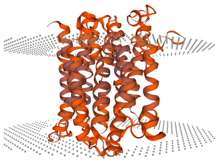 |
| PtrMFS34 | POPTR_014G085700v3 | LOC7496975 | 6v4d.1.A | 37.25% | Vesicular glutamate transporter 2. Structure of the rat vesicular glutamate transporter 2 determined by single particle Cryo-EM. | 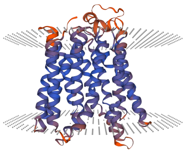 |

| | | | | | | |
|---|---|---|---|---|---|---|
| PtrMFS35 | POPTR_015G067000v3 | LOC7462526 | 6g9x.1.A | 17.50% | Major facilitator superfamily MFS_1. Crystal structure of a MFS transporter at 2.54 Angstroem resolution. | 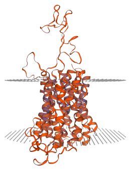 |
| PtrMFS36 | POPTR_015G081300v3 | LOC7457762 | 7cko.1.A | 11.02% | Monocarboxylate transporter 1. Cryo-EM structure of the human MCT1/Basigin-2 complex in the presence of anti-cancer drug candidate 7ACC2 in the inward-open conformation. | 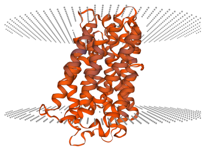 |
| PtrMFS37 | POPTR_015G081500v3 | LOC7457764 | 1pw4.1.A | 18.09% | Glycerol-3-phosphate transporter. Crystal Structure of the Glycerol-3-Phosphate Transporter from E.Coli. | 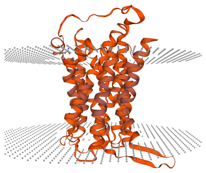 |
| PtrMFS38 | POPTR_016G024400v3 | LOC7455882 | 6s4m.1.A | 19.31% | Major facilitator superfamily domain-containing protein 10. Crystal structure of the human organic anion transporter MFSD10 (TETRAN). | 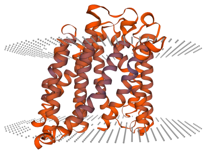 |
| PtrMFS39 | POPTR_016G111000v3 | LOC7466045 | 6v4d.1.A | 30.15% | Vesicular glutamate transporter 2. Structure of the rat vesicular glutamate transporter 2 determined by single particle Cryo-EM. | 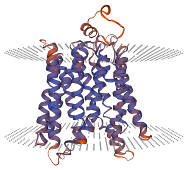 |
| PtrMFS40 | POPTR_018G115000v3 | LOC7489481 | 1pw4.1.A | 21.22% | Glycerol-3-phosphate transporter. Crystal Structure of the Glycerol-3-Phosphate Transporter from E.Coli. | 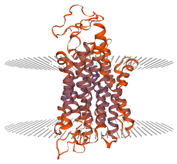 |
| PtrMFS41 | POPTR_018G121600v3 | LOC18111057 | 6v4d.1.A | 30.07% | Vesicular glutamate transporter 2. Structure of the rat vesicular glutamate transporter 2 determined by single particle Cryo-EM. | 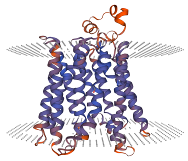 |
