# Supplementary material for: Genome-Wide Analysis of Major Facilitator Superfamily and Its Expression in Response of Poplar to Fusarium oxysporum
Source: Front Genet. 2021 Oct 22;12:769888. doi: 10.3389/fgene.2021.769888 (PMC8567078; doi:10.3389/fgene.2021.769888)
Supplement: Supplementary file 10 [file Table1.DOC]

**Table S1**. The components of the qRT-PCR assays

| **Reaction solution** | **Volume（μL）** |
| --- | --- |
| SYBR Green Realtime PCR Master Mix | 10 |
| cDNA | 2 |
| Primer F（10 μM） | 1 |
| Primer R（10 μM） | 1 |
| dd H_2_O | 6 |
