# Supplementary material for: Genome-Wide Analysis of Major Facilitator Superfamily and Its Expression in Response of Poplar to Fusarium oxysporum
Source: Front Genet. 2021 Oct 22;12:769888. doi: 10.3389/fgene.2021.769888 (PMC8567078; doi:10.3389/fgene.2021.769888)
Supplement: Supplementary file 11 [file Table5.DOC]

**Table S8**. Subcellular localization and gene coordinate of PtrMFS proteins

| **Name in this paper** | **Gene Name** | **Locus tag** | **Subcellular localization** |
| --- | --- | --- | --- |
| PtrMFS1 | LOC7478080 | POPTR_001G111400v3 | Plasma membrane |
| PtrMFS2 | LOC18094006 | POPTR_001G124200v3 | Plasma membrane |
| PtrMFS3 | LOC18094568 | POPTR_001G152300v3 | Plasma membrane |
| PtrMFS4 | LOC7468011 | POPTR_001G248200v3 | chloroplast |
| PtrMFS5 | LOC7478542 | POPTR_001G249800v3 | chloroplast |
| PtrMFS6 | LOC7470780 | POPTR_001G286600v3 | Plasma membrane |
| PtrMFS7 | LOC7487860 | POPTR_001G348300v3 | Plasma membrane |
| PtrMFS8 | LOC7496897 | POPTR_002G016200v3 | Plasma membrane |
| PtrMFS9 | LOC18096251 | POPTR_002G106900v3 | Plasma membrane |
| PtrMFS10 | LOC18096883 | POPTR_003G082400v3 | Plasma membrane |
| PtrMFS11 | LOC7465506 | POPTR_003G109300v3 | Plasma membrane |
| PtrMFS12 | LOC7479251 | POPTR_003G120600v3 | Plasma membrane |
| PtrMFS13 | LOC7453664 | POPTR_004G178600v3 | chloroplast |
| PtrMFS14 | LOC7494001 | POPTR_005G245900v3 | Plasma membrane |
| PtrMFS15 | LOC7479627 | POPTR_006G026200v3 | Plasma membrane |
| PtrMFS16 | LOC18099899 | POPTR_006G062300v3 | Plasma membrane |
| PtrMFS17 | LOC7497754 | POPTR_007G003100v3 | Plasma membrane |
| PtrMFS18 | LOC7473146 | POPTR_007G030800v3 | Plasma membrane |
| PtrMFS19 | LOC18100946 | POPTR_007G091700v3 | Plasma membrane |
| PtrMFS20 | LOC7490218 | POPTR_007G091800v3 | Plasma membrane |
| PtrMFS21 | LOC7486851 | POPTR_008G010600v3 | Plasma membrane |
| PtrMFS22 | LOC7486914 | POPTR_008G022100v3 | chloroplast |
| PtrMFS23 | LOC7463316 | POPTR_009G006400v3 | Plasma membrane |
| PtrMFS24 | LOC7463308 | POPTR_009G008500v3 | Plasma membrane |
| PtrMFS25 | LOC7463307 | POPTR_009G008600v3 | Plasma membrane |
| PtrMFS26 | LOC7488173 | POPTR_009G021700v3 | Endoplasmic reticulum |
| PtrMFS27 | LOC7481487 | POPTR_009G043800v3 | Plasma membrane |
| PtrMFS28 | LOC7478769 | POPTR_009G081100v3 | Plasma membrane |
| PtrMFS29 | LOC7474984 | POPTR_009G138900v3 | chloroplast |
| PtrMFS30 | LOC7463360 | POPTR_009G168200v3 | Plasma membrane |
| PtrMFS31 | LOC7477174 | POPTR_010G237300v3 | vacuole |
| PtrMFS32 | LOC7458066 | POPTR_012G087700v3 | Plasma membrane |
| PtrMFS33 | LOC7496936 | POPTR_014G078000v3 | Plasma membrane |
| PtrMFS34 | LOC7496975 | POPTR_014G085700v3 | Plasma membrane |
| PtrMFS35 | LOC7462526 | POPTR_015G067000v3 | Plasma membrane |
| PtrMFS36 | LOC7457762 | POPTR_015G081300v3 | Plasma membrane |
| PtrMFS37 | LOC7457764 | POPTR_015G081500v3 | Plasma membrane |
| PtrMFS38 | LOC7455882 | POPTR_016G024400v3 | Plasma membrane |
| PtrMFS39 | LOC7466045 | POPTR_016G111000v3 | chloroplast |
| PtrMFS40 | LOC7489481 | POPTR_018G115000v3 | Plasma membrane |
| PtrMFS41 | LOC18111057 | POPTR_018G121600v3 | chloroplast |
