# Supplementary material for: Genome-Wide Analysis of Major Facilitator Superfamily and Its Expression in Response of Poplar to Fusarium oxysporum
Source: Front Genet. 2021 Oct 22;12:769888. doi: 10.3389/fgene.2021.769888 (PMC8567078; doi:10.3389/fgene.2021.769888)
Supplement: Supplementary file 12 [file Table2.DOCX]

**Table S2**. Primer Sequences of qRT-PCR

| **Gene** | **Forward Primers (5'-3')** | **Reverse Primers (5'-3')** |
| --- | --- | --- |
| *Pdpapactin* | GCTGAGAGATTCCGTTGCCCTG | GGCGGTGATCTCCTTGCTCATT |
| *PdpapEF1-α* | TGGGTCGTGTTGAAACTGGTGT | GGCAGGATCGTCCTTGGAGTTC |
| *PdpapMFS1* | GCCGTGTTTTCTGTGGATTG | CCTGGCAAAGTGTCTTGATTG |
| *PdpapMFS2* | TGTATTGTTCCATCCCTGCTC | AGCCTGTTCCGTCTATGATTG |
| *PdpapMFS3* | CCCAGTAGCATAGTCAAGAGTG | CAGCACCAAACATTCCCAAAG |
| *PdpapMFS4* | GTTAAACGAAGATTGCGAGCC | TCCAACCATGAGCAAGAGAC |
| *PdpapMFS5* | TCCTTGTCGCCCTCTAAAATC | AACATCAGCATACACCTTCCC |
| *PdpapMFS6* | CGTATATGGTGGACTGGTTGG | TTGTCTCTATGGATGTCAGCG |
| *PdpapMFS7* | GATAGCAGGGATGACAGGAAG | AGGGAAGTAGATGAGGGTGAC |
| *PdpapMFS8* | TGATCCCTGCCATTACTGATG | AGCTTTCCGACCATACTTGTC |
| *PdpapMFS9* | GTGTTTGGGTTTTGGATTCTCG | AGGCAGACTTGAGATTCGC |
| *PdpapMFS10* | CCCAGTAGCATAGTCAAGAGTG | TGACCAGCACCATACATTCC |
| *PdpapMFS11* | GTTTTGCCTGGTCTCGTTATTG | TTAACATCAGGGTCCGAAGC |
| *PdpapMFS12* | TTTCTGTGGATTGGGCTCTG | CAAGCCAGAGACATAACCCAG |
| *PdpapMFS13* | TTCCTCTCCACTGCTTTAACG | GCTTGCGAAAATGTTACCCTG |
| *PdpapMFS14* | CTTTCCACTCGTTTCCTCTCC | TCCTTTTCCTTTCGGTTTCCC |
| *PdpapMFS15* | TTTCCATACCTCTTACCTTGCC | TTTCAGTAGGCTTTTCCGAGG |
| *PdpapMFS16* | AACCTCACTCTTGAACACGAC | AAAACCCACTTCACTAGACCC |
| *PdpapMFS17* | GTCGTAAACTTTCAATGGCGG | CCCATACCTGTTGATCTCACTG |
| *PdpapMFS18* | TTATAAACCATGCCCGTCCC | CAAATCCAGTACCCCTCACAC |
| *PdpapMFS19* | ATTCGGTAGTCTGTTTGGAGG | GACCATGCTTGAATGCTGTTG |
| *PdpapMFS20* | CTCTTGGTGCTTTTCTTTGGG | GAACAGAGCCCTCCGATAATG |
| *PdpapMFS21* | CTGAGCGAGAGGAAGATATTGG | GAGTATTGAAAATGACCACTGCG |
| *PdpapMFS22* | GTTTGAGAGCCCTACACTTAGAG | TTGCACCGATTCCTACCATC |
| *PdpapMFS23* | CATATCTCTTTGGCACCTACTCC | CCAACCACAAGCACAAACC |
| *PdpapMFS25* | ACCCCTTGTTCCTATCATTCG | TCAGAAACGCACACCCATATC |
| *PdpapMFS26* | GCCAATATGTTCACTGTTCCC | TGAGCACAACCACTGAAGAG |
| *PdpapMFS27* | CTTCTCTACTCTTTCACCGACC | CTCCAATATCTCATACGGCTCC |
| *PdpapMFS28* | CGCTGACATCCATAGAGACTG | AACCTTCTCAAGCAAAAGCAC |
| *PdpapMFS29* | CAGCTATGATCTGTGAAGGGAG | TCGCTTTGTGGTAGACTTTCC |
| *PdpapMFS30* | CCATGAAAGGTCTAGATCCGTG | TGAGTGGCAGTAAAGATTCCC |
| *PdpapMFS31* | ACTTCTGCTTTATGTCCTGGTC | GTACACATACACAGCATCCCTAG |
| *PdpapMFS32* | TGACCTCAAACTCCATACAGC | TCCCAAGGTTTGCACTATCC |
| *PdpapMFS33* | CACTTCTATCTCGGGTCATGTC | AGTTGTAGGTGAAGCAGGTG |
| *PdpapMFS34* | TGCCATACTTCCCATGTCAC | TCTCCAATCCCCATGAAAGC |
| *PdpapMFS35* | TCCTTTACTCCGCTTTCACTC | AGAAACACATCACCGCCAC |
| *PdpapMFS36* | GGATTCAGAACACAAGGCAAC | GTCACAAGCAGTCCCCATAG |
| *PdpapMFS37* | GGATTCAGAACACAAGGCAAC | AAAGAGGTCACAAGCAGTCC |
| *PdpapMFS38* | ATGCTGGTTATGTGGGATCTG | ACCTCGTAGAAATTGCCATCC |
| *PdpapMFS39* | CTTCCTCACCTAACACCTCTTC | CTTGCTTCACCTTTTCCCTTG |
| *PdpapMFS40* | TGGAGAATTTGGAGAAGGTGG | TGTGACTTATGTAGAAGGGCAAC |
| *PdpapMFS41* | CTACTTCACGGTCCCAAACTATC | AGGATAGAAACCCATGCAGC |
