# Supplementary material for: Genome-Wide Analysis of Major Facilitator Superfamily and Its Expression in Response of Poplar to Fusarium oxysporum
Source: Front Genet. 2021 Oct 22;12:769888. doi: 10.3389/fgene.2021.769888 (PMC8567078; doi:10.3389/fgene.2021.769888)
Supplement: Supplementary file 13 [file Table3.DOCX]

**Table S3**. Physicochemical properties of PtrMFSs proteins

| **Name in this paper** | **Gene ID** | **Locus tag** | **Number of amino acids** | **Molecular weight (kDa)** | **Theoretical pI** | **Aliphatic index** | **Grand average of hydropathicity (GRAVY)** | **Formula** | **Total number of atoms** | **Instability index** | **stability** |
| --- | --- | --- | --- | --- | --- | --- | --- | --- | --- | --- | --- |
| PtrMFS1 | LOC7478080 | POPTR_001G111400v3 | 697 | 77.81 | 5.96 | 107.83 | 0.223 | C_3,542_H_5,559_N_901_O_1,013_S_26_ | 11,041 | 39.72 | Stable |
| PtrMFS2 | LOC18094006 | POPTR_001G124200v3 | 523 | 55.99 | 8.57 | 103.96 | 0.440 | C_2,588_H_4,000_N_646_O_711_S_14_ | 7,959 | 31.56 | Stable |
| PtrMFS3 | LOC18094568 | POPTR_001G152300v3 | 537 | 57.94 | 8.99 | 98.64 | 0.344 | C_2,653_H_4,125_N_677_O_729_S_24_ | 8,208 | 34.82 | Stable |
| PtrMFS4 | LOC7468011 | POPTR_001G248200v3 | 528 | 57.99 | 10.07 | 98.83 | 0.396 | C_2,680_H_4,131_N_689_O_705_S_22_ | 8,227 | 38.25 | Stable |
| PtrMFS5 | LOC7478542 | POPTR_001G249800v3 | 390 | 42.27 | 8.59 | 100.74 | 0.494 | C_1,973_H_2,988_N_478_O_524_S_15_ | 5,978 | 42.30 | Unstable |
| PtrMFS6 | LOC7470780 | POPTR_001G286600v3 | 502 | 54.10 | 6.92 | 100.22 | 0.552 | C_2,497_H_3,839_N_605_O_687_S_24_ | 7,652 | 27.36 | Stable |
| PtrMFS7 | LOC7487860 | POPTR_001G348300v3 | 471 | 50.79 | 8.87 | 98.98 | 0.533 | C_2,322_H_3,606_N_580_O_636_S_30_ | 7,174 | 42.96 | Unstable |
| PtrMFS8 | LOC7496897 | POPTR_002G016200v3 | 450 | 48.73 | 8.76 | 102.96 | 0.652 | C_2,231_H_3,491_N_545_O_612_S_31_ | 6,910 | 37.65 | Stable |
| PtrMFS9 | LOC18096251 | POPTR_002G106900v3 | 468 | 51.61 | 8.98 | 101.69 | 0.406 | C_2,366_H_3,642_N_618_O_625_S_27_ | 7,278 | 52.06 | Unstable |
| PtrMFS10 | LOC18096883 | POPTR_003G082400v3 | 534 | 57.64 | 9.20 | 101.39 | 0.395 | - | - | 37.53 | Stable |
| PtrMFS11 | LOC7465506 | POPTR_003G109300v3 | 522 | 55.89 | 8.55 | 105.06 | 0.452 | C_2,576_H_3,994_N_650_O_709_S_15_ | 7,944 | 38.17 | Stable |
| PtrMFS12 | LOC7479251 | POPTR_003G120600v3 | 698 | 77.98 | 5.96 | 105.03 | 0.183 | C_3,542_H_5,564_N_898_O_1,018_S_30_ | 11,052 | 41.92 | Unstable |
| PtrMFS13 | LOC7453664 | POPTR_004G178600v3 | 372 | 40.20 | 8.81 | 108.39 | 0.618 | C_1,848_H_2,896_N_460_O_500_S_20_ | 5,724 | 38.00 | Stable |
| PtrMFS14 | LOC7494001 | POPTR_005G245900v3 | 447 | 48.30 | 8.76 | 109.78 | 0.701 | C_2,219_H_3,487_N_543_O_602_S_28_ | 6,879 | 35.45 | Stable |
| PtrMFS15 | LOC7479627 | POPTR_006G026200v3 | 415 | 45.19 | 8.73 | 110.48 | 0.625 | C_2,090_H_3,250_N_516_O_553_S_23_ | 6,432 | 34.03 | Stable |
| PtrMFS16 | LOC18099899 | POPTR_006G062300v3 | 539 | 58.55 | 7.61 | 95.92 | 0.301 | C_2,700_H_4,117_N_675_O_750_S_16_ | 8,258 | 34.23 | Stable |
| PtrMFS17 | LOC7497754 | POPTR_007G003100v3 | 529 | 58.23 | 8.96 | 103.21 | 0.426 | C_2,684_H_4,194_N_672_O_712_S_30_ | 8,292 | 41.86 | Unstable |
| PtrMFS18 | LOC7473146 | POPTR_007G030800v3 | 496 | 54.26 | 9.34 | 97.18 | 0.437 | C_2,493_H_3,855_N_633_O_669_S_27_ | 7,677 | 25.81 | Stable |
| PtrMFS19 | LOC18100946 | POPTR_007G091700v3 | 513 | 55.65 | 5.54 | 103.06 | 0.277 | C_2,531_H_3,962_N_644_O_733_S_16_ | 7,886 | 34.16 | Stable |
| PtrMFS20 | LOC7490218 | POPTR_007G091800v3 | 426 | 46.38 | 5.65 | 98.92 | 0.202 | C_2,091_H_3,297_N_543_O_610_S_18_ | 6,559 | 27.96 | Stable |
| PtrMFS21 | LOC7486851 | POPTR_008G010600v3 | 453 | 49.55 | 7.57 | 107.04 | 0.370 | C_2,274_H_3,557_N_571_O_630_S_18_ | 7,050 | 25.98 | Stable |
| PtrMFS22 | LOC7486914 | POPTR_008G022100v3 | 373 | 40.98 | 6.52 | 109.60 | 0.568 | C_1,904_H_2,924_N_458_O_519_S_14_ | 5,819 | 44.53 | Unstable |
| PtrMFS23 | LOC7463316 | POPTR_009G006400v3 | 559 | 61.66 | 9.04 | 109.71 | 0.562 | C_2,870_H_4,487_N_685_O_759_S_29_ | 8,830 | 26.13 | Stable |
| PtrMFS24 | LOC7463308 | POPTR_009G008500v3 | 530 | 57.25 | 9.18 | 85.96 | 0.355 | C_2,616_H_4,003_N_667_O_718_S_30_ | 8,034 | 37.60 | Stable |
| PtrMFS25 | LOC7463307 | POPTR_009G008600v3 | 530 | 57.19 | 9.11 | 86.34 | 0.364 | C_2,617_H_3,995_N_665_O_718_S_29_ | 8,024 | 38.46 | Stable |
| PtrMFS26 | LOC7488173 | POPTR_009G021700v3 | 459 | 50.41 | 7.55 | 111.31 | 0.525 | C_2,326_H_3,628_N_580_O_632_S_18_ | 7,184 | 43.57 | Unstable |
| PtrMFS27 | LOC7481487 | POPTR_009G043800v3 | 520 | 57.57 | 8.52 | 96.71 | 0.293 | C_2,661_H_4,060_N_670_O_716_S_21_ | 8,128 | 44.57 | Unstable |
| PtrMFS28 | LOC7478769 | POPTR_009G081100v3 | 429 | 46.42 | 7.49 | 107.27 | 0.706 | C_2,164_H_3,316_N_516_O_570_S_23_ | 6,589 | 31.39 | Stable |
| PtrMFS29 | LOC7474984 | POPTR_009G138900v3 | 384 | 41.32 | 8.56 | 118.46 | 0.714 | C_1,902_H_3,020_N_462_O_520_S_20_ | 5,924 | 41.70 | Unstable |
| PtrMFS30 | LOC7463360 | POPTR_009G168200v3 | 442 | 48.30 | 9.76 | 106.99 | 0.565 | C_2,240_H_3,485_N_567_O_583_S_19_ | 6,894 | 41.59 | Unstable |
| PtrMFS31 | LOC7477174 | POPTR_010G237300v3 | 374 | 40.96 | 8.11 | 117.83 | 0.758 | C_1,913_H_2,976_N_448_O_512_S_16_ | 5,865 | 40.73 | unstable |
| PtrMFS32 | LOC7458066 | POPTR_012G087700v3 | 508 | 54.90 | 9.01 | 93.39 | 0.412 | C_2,529_H_3,884_N_644_O_684_S_20_ | 7,761 | 38.35 | Stable |
| PtrMFS33 | LOC7496936 | POPTR_014G078000v3 | 697 | 78.23 | 6.40 | 109.91 | 0.208 | C_3,574_H_5,589_N_913_O_1,013_S_21_ | 11,110 | 35.02 | Stable |
| PtrMFS34 | LOC7496975 | POPTR_014G085700v3 | 574 | 62.96 | 9.36 | 97.87 | 0.314 | C_2,887_H_4,458_N_748_O_776_S_28_ | 8,897 | 44.73 | Unstable |
| PtrMFS35 | LOC7462526 | POPTR_015G067000v3 | 551 | 60.02 | 8.80 | 109.36 | 0.559 | C_2,744_H_4,288_N_698_O_760_S_25_ | 8,515 | 34.87 | Stable |
| PtrMFS36 | LOC7457762 | POPTR_015G081300v3 | 508 | 54.90 | 9.02 | 92.58 | 0.396 | C_2,514_H_3,878_N_646_O_686_S_24_ | 7,748 | 38.33 | Stable |
| PtrMFS37 | LOC7457764 | POPTR_015G081500v3 | 508 | 54.91 | 9.10 | 90.65 | 0.388 | C_2,514_H_3,876_N_646_O_685_S_25_ | 7,746 | 38.59 | Stable |
| PtrMFS38 | LOC7455882 | POPTR_016G024400v3 | 403 | 44.09 | 8.88 | 113.00 | 0.645 | C_2,045_H_3,185_N_501_O_541_S_20_ | 6,292 | 36.43 | Stable |
| PtrMFS39 | LOC7466045 | POPTR_016G111000v3 | 513 | 55.52 | 9.96 | 99.65 | 0.428 | C_2,549_H_3,967_N_663_O_686_S_20_ | 7,885 | 38.59 | Stable |
| PtrMFS40 | LOC7489481 | POPTR_018G115000v3 | 506 | 54.26 | 6.31 | 115.57 | 0.663 | C_2,531_H_3,916_N_616_O_679_S_13_ | 7,755 | 24.80 | Stable |
| PtrMFS41 | LOC18111057 | POPTR_018G121600v3 | 539 | 58.38 | 7.56 | 97.90 | 0.334 | C_2,699_H_4,130_N_664_O_751_S_15_ | 8,259 | 33.21 | Stable |
