# Supplementary material for: Genome-Wide Analysis of Major Facilitator Superfamily and Its Expression in Response of Poplar to Fusarium oxysporum
Source: Front Genet. 2021 Oct 22;12:769888. doi: 10.3389/fgene.2021.769888 (PMC8567078; doi:10.3389/fgene.2021.769888)
Supplement: Supplementary file 14 [file Table4.DOCX]

**Table S5**. Predition of the Sencondary Structure in PtrMFS Proteins

| **Name in this paper** | **Gene Name** | **Number of Alpha helix** | **The percent of all the sencondary structures** | **Number of Extended strand** | **The percent of all the sencondary structures** | **Number of Beta turn** | **The percent of all the sencondary structures** | **Number of Random coil** | **The percent of all the sencondary structures** |
| --- | --- | --- | --- | --- | --- | --- | --- | --- | --- |
| PtrMFS1 | LOC7478080 | 446 | 63.99% | 63 | 9.04% | 21 | 3.01% | 167 | 23.96% |
| PtrMFS2 | LOC18094006 | 229 | 43.79% | 67 | 12.81% | 26 | 4.97% | 201 | 38.43% |
| PtrMFS3 | LOC18094568 | 216 | 40.22% | 70 | 13.04% | 27 | 5.03% | 224 | 41.71% |
| PtrMFS4 | LOC7468011 | 206 | 39.02% | 93 | 17.61% | 32 | 6.06% | 197 | 37.31% |
| PtrMFS5 | LOC7478542 | 158 | 40.51% | 82 | 21.03% | 31 | 7.95% | 119 | 30.51% |
| PtrMFS6 | LOC7470780 | 212 | 42.23% | 93 | 18.53% | 24 | 4.78% | 173 | 34.46% |
| PtrMFS7 | LOC7487860 | 223 | 47.35% | 75 | 15.92% | 26 | 5.52% | 147 | 31.21% |
| PtrMFS8 | LOC7496897 | 229 | 50.89% | 56 | 12.44% | 21 | 4.67% | 144 | 32.00% |
| PtrMFS9 | LOC18096251 | 214 | 45.73% | 69 | 14.74% | 14 | 2.99% | 171 | 36.54% |
| PtrMFS10 | LOC18096883 | 216 | 40.45% | 78 | 14.61% | 26 | 4.87% | 214 | 40.07% |
| PtrMFS11 | LOC7465506 | 229 | 43.87% | 66 | 12.64% | 28 | 5.36% | 199 | 38.12% |
| PtrMFS12 | LOC7479251 | 435 | 62.32% | 71 | 10.17% | 21 | 3.01% | 171 | 24.50% |
| PtrMFS13 | LOC7453664 | 184 | 49.46% | 49 | 13.17% | 17 | 4.57% | 122 | 32.80% |
| PtrMFS14 | LOC7494001 | 239 | 53.47% | 52 | 11.63% | 15 | 3.36% | 141 | 31.54% |
| PtrMFS15 | LOC7479627 | 200 | 48.19% | 55 | 13.25% | 15 | 3.61% | 145 | 34.94% |
| PtrMFS16 | LOC18099899 | 216 | 40.07% | 106 | 19.67% | 21 | 3.90% | 196 | 36.36% |
| PtrMFS17 | LOC7497754 | 243 | 45.94% | 80 | 15.12% | 24 | 4.54% | 182 | 34.40% |
| PtrMFS18 | LOC7473146 | 257 | 51.81% | 62 | 12.50% | 15 | 3.02% | 162 | 32.66% |
| PtrMFS19 | LOC18100946 | 284 | 55.36% | 64 | 12.48% | 20 | 3.90% | 145 | 28.27% |
| PtrMFS20 | LOC7490218 | 211 | 49.53% | 57 | 13.38% | 19 | 4.46% | 139 | 32.63% |
| PtrMFS21 | LOC7486851 | 230 | 50.77% | 68 | 15.01% | 23 | 5.08% | 132 | 29.14% |
| PtrMFS22 | LOC7486914 | 188 | 50.40% | 52 | 13.94% | 18 | 4.83% | 115 | 30.83% |
| PtrMFS23 | LOC7463316 | 256 | 45.80% | 102 | 18.25% | 23 | 4.11% | 178 | 31.84% |
| PtrMFS24 | LOC7463308 | 223 | 42.08% | 92 | 17.36% | 34 | 6.42% | 181 | 34.15% |
| PtrMFS25 | LOC7463307 | 232 | 43.77% | 86 | 16.23% | 37 | 6.98% | 175 | 33.02% |
| PtrMFS26 | LOC7488173 | 240 | 52.29% | 51 | 11.11% | 16 | 3.49% | 152 | 33.12% |
| PtrMFS27 | LOC7481487 | 203 | 39.04% | 99 | 19.04% | 21 | 4.04% | 197 | 37.88% |
| PtrMFS28 | LOC7478769 | 184 | 42.89% | 95 | 22.14% | 12 | 2.80% | 138 | 32.17% |
| PtrMFS29 | LOC7474984 | 195 | 50.78% | 54 | 14.06% | 13 | 3.39% | 122 | 31.77% |
| PtrMFS30 | LOC7463360 | 205 | 46.38% | 83 | 18.78% | 31 | 7.01% | 123 | 27.83% |
| PtrMFS31 | LOC7477174 | 201 | 53.74% | 48 | 12.83% | 19 | 5.08% | 106 | 28.34% |
| PtrMFS32 | LOC7458066 | 229 | 45.08% | 80 | 15.75% | 30 | 5.91% | 169 | 33.27% |
| PtrMFS33 | LOC7496936 | 428 | 61.41% | 80 | 11.48% | 22 | 3.16% | 167 | 23.96% |
| PtrMFS34 | LOC7496975 | 200 | 34.84% | 100 | 17.42% | 31 | 5.40% | 243 | 42.33% |
| PtrMFS35 | LOC7462526 | 251 | 45.55% | 102 | 18.51% | 24 | 4.36% | 174 | 31.58% |
| PtrMFS36 | LOC7457762 | 234 | 46.06% | 74 | 14.57% | 28 | 5.51% | 172 | 33.86% |
| PtrMFS37 | LOC7457764 | 232 | 45.67% | 74 | 14.57% | 34 | 6.69% | 168 | 33.07% |
| PtrMFS38 | LOC7455882 | 201 | 49.88% | 50 | 12.41% | 16 | 3.97% | 136 | 33.75% |
| PtrMFS39 | LOC7466045 | 221 | 43.08% | 91 | 17.74% | 28 | 5.46% | 173 | 33.72% |
| PtrMFS40 | LOC7489481 | 224 | 44.27% | 68 | 13.44% | 23 | 4.55% | 191 | 37.75% |
| PtrMFS41 | LOC18111057 | 194 | 35.99% | 107 | 19.85% | 29 | 5.38% | 209 | 38.78% |
