# Supplementary material for: Genome-Wide Analysis of Major Facilitator Superfamily and Its Expression in Response of Poplar to Fusarium oxysporum
Source: Front Genet. 2021 Oct 22;12:769888. doi: 10.3389/fgene.2021.769888 (PMC8567078; doi:10.3389/fgene.2021.769888)
Supplement: Supplementary file 15 [file Table11.DOCX]

**Table S14**. Gene ontology analysis

| **GO term** | **Ontology** | **Description** | **Number in input list** | **Number in BG/Ref** | **p-value** | **FDR** |
| --- | --- | --- | --- | --- | --- | --- |
| GO:0055085 | Biological Process | transmembrane transport | 41 | 837 | 3.20E-58 | 3.70E-57 |
| GO:0051234 | Biological Process | establishment of localization | 41 | 1756 | 3.00E-45 | 1.10E-44 |
| GO:0006810 | Biological Process | transport | 41 | 1756 | 3.00E-45 | 1.10E-44 |
| GO:0051179 | Biological Process | localization | 41 | 1778 | 5.00E-45 | 1.40E-44 |
| GO:0009987 | Biological Process | cellular process | 41 | 9774 | 7.40E-15 | 1.70E-14 |
| GO:0005215 | Molecular Function | transporter activity | 12 | 1148 | 9.70E-07 | 2.90E-06 |
| GO:0022857 | Molecular Function | transmembrane transporter activity | 10 | 866 | 4.00E-06 | 5.90E-06 |
| GO:0016021 | Cellular Component | integral to membrane | 41 | 1121 | 4.00E-53 | 5.90E-52 |
| GO:0031224 | Cellular Component | intrinsic to membrane | 41 | 1337 | 4.90E-50 | 3.60E-49 |
| GO:0044425 | Cellular Component | membrane part | 41 | 1613 | 9.60E-47 | 4.70E-46 |
| GO:0016020 | Cellular Component | membrane | 41 | 3013 | 1.00E-35 | 3.70E-35 |
| GO:0044464 | Cellular Component | cell part | 41 | 5868 | 6.50E-24 | 1.60E-23 |
| GO:0005623 | Cellular Component | cell | 41 | 5868 | 6.50E-24 | 1.60E-23 |
