# Supplementary material for: Genome-Wide Analysis of Major Facilitator Superfamily and Its Expression in Response of Poplar to Fusarium oxysporum
Source: Front Genet. 2021 Oct 22;12:769888. doi: 10.3389/fgene.2021.769888 (PMC8567078; doi:10.3389/fgene.2021.769888)
Supplement: Supplementary file 16 [file Table12.DOC]

Statement of resubmission

**Reasons for rejection:**

Objective errors in the methods, applications, or interpretations were identified in this manuscript that prevent further consideration.

**Comments of editor:**

Key deficiencies in the manuscript make its review premature. Once these are fully addressed, it can be resubmitted for consideration.

**Response to editor:**

Dear editor ,

I am really sorry that the previous manuscript has objective errors and didn’t meet the publication requirements. To make the manuscript meet the publication requirements of 《Frontiers in Genetics》 to the maximum, I have carefully checked and revised the full manuscript. I have made a comprehensive adjustment to the methods and expressions involved in the manuscript. The adjusted parts have been marked in red in the article. After correcting the errors in the manuscript, I have resubmitted the article in the submission system. I sincerely hope that the revised manuscript can be reconsidered by you.

Thank you for the precious opportunity and wish you success in your work!
